# Pan-cancer analyses suggest kindlin-associated global mechanochemical alterations

**DOI:** 10.1038/s42003-024-06044-5

**Published:** 2024-03-28

**Authors:** Debojyoti Chowdhury, Ayush Mistry, Debashruti Maity, Riti Bhatia, Shreyansh Priyadarshi, Simran Wadan, Soham Chakraborty, Shubhasis Haldar

**Affiliations:** 1 Department of Chemical and Biological Sciences, S.N. Bose National Centre for Basic Sciences, Kolkata, West Bengal 700106 India; 2 Department of Biology, Trivedi School of Biosciences, Ashoka University, Sonepat, Haryana 131029 India; 3 Technical Research Centre, S.N. Bose National Centre for Basic Sciences, Kolkata, West Bengal 700106 India

**Keywords:** Cancer genomics, Cellular signalling networks, Data processing

## Abstract

Kindlins serve as mechanosensitive adapters, transducing extracellular mechanical cues to intracellular biochemical signals and thus, their perturbations potentially lead to cancer progressions. Despite the kindlin involvement in tumor development, understanding their genetic and mechanochemical characteristics across different cancers remains elusive. Here, we thoroughly examined genetic alterations in kindlins across more than 10,000 patients with 33 cancer types. Our findings reveal cancer-specific alterations, particularly prevalent in advanced tumor stage and during metastatic onset. We observed a significant co-alteration between kindlins and mechanochemical proteome in various tumors through the activation of cancer-related pathways and adverse survival outcomes. Leveraging normal mode analysis, we predicted structural consequences of cancer-specific kindlin mutations, highlighting potential impacts on stability and downstream signaling pathways. Our study unraveled alterations in epithelial–mesenchymal transition markers associated with kindlin activity. This comprehensive analysis provides a resource for guiding future mechanistic investigations and therapeutic strategies targeting the roles of kindlins in cancer treatment.

## Abstract (web summary)

Pan-cancer analysis of kindlin genes on patient genomic data reveals a distinct pattern of kindlin activity. Our analysis suggests kindlins as mechanochemical connector of major cancer hallmark pathways.

## Introduction

Cancer poses an ever-increasing threat and is projected to become more dead in the coming years^1,2^. The severity of this multifaceted disease arises from its ability to enable malignant cells to migrate swiftly, protrude into tissues, invade, metastasize, and resist chemotherapy^3,4^. This complex interplay of processes is influenced by numerous factors, including mechanical forces originating from the extracellular matrix (ECM)^5^. These crucial cell-ECM interactions are majorly mediated through specialized structures known as focal adhesions as well as hemidesmosomes, dystroglycan complex, and syndecans, which act as mechanosensory hubs that translate external mechanical cues into intracellular rearrangements and chemical signals^6–8^. Among the myriad of proteins participating in these intricate processes, the kindlin family of mechanosensing adapter proteins has emerged as a crucial player^9,10^. The kindlin family of FERM domain-containing proteins comprises three members, kindlin 1, 2, and 3, which are encoded by the *FERMT1*, *FERMT2*, and *FERMT3* genes, respectively. Kindlins play a pivotal role in conveying extracellular signals by physically interacting with structural proteins, receptors, and transcription factors, ultimately triggering a cascade of chemical responses within cells^11–13^. Notably, these proteins are closely linked to virtually every facet of cancer biology, influencing tumor-microenvironment interactions, cellular metabolism, cell cycle progression, transcriptional regulation, and even the regulation of cancer stem cells^14–17^.

In recent years, the role of kindlins in cancer has gained attention for two main reasons. First, kindlins act as adapter proteins to connect multiple cancer-promoting pathways, in addition to their known role in integrin activation^12,13^. For example, experiments have revealed that kindlin2 can regulate Hippo signaling by modulating the nuclear localization of YAP^18^. Additionally, kindlins regulate breast cancer growth and metastasis through the TGFβ/EGF signaling axis^19^. It also influences cancer cell stemness via the Wnt/beta-catenin and Hedgehog pathways^20,21^. Kindlin1, on the other hand, independently regulates IL-6 secretion and hence the immune microenvironment in breast cancer^22^. Cancer-specific metabolic regulation, such as proline biosynthesis,is also governed by Kindlin2^16^. Furthermore, all of these kindlins are involved in growth factor induction, tumor promotion and angiogenesis^23,24^. With mounting evidence supporting the involvement of kindlin family proteins in numerous cancer-associated pathways, further exploration of their roles is imperative.Structural disruptions in these proteins could have a global impact on mechanochemical signaling, leading to disruptions in mechanical homeostasis^25^. Second, as a mechanosensitive adapter protein, kindlin connects extracellular mechanical cues with intracellular chemical events^26^. Therefore, understanding the role of kindlins in cancer will help us decipher the intricate interplay between tumors and their microenvironment. This approach will be crucial for developing precision therapy for cancer treatment, especially for overcoming chemoresistance and cancer recurrence.Given their extensive involvement in various cancer-associated pathways, there is a compelling need to delve deeper into the roles of kindlin family proteins in cancer. Mutations in these proteins can significantly impact their mechanochemical signaling capabilities, potentially disrupting global mechanical homeostasis within cells^27–29^. Understanding the consequences of such genetic alterations, especially in mechanosensitive proteins such as kindlins, is essential for unraveling the intricate mechanisms underpinning cancer progression.

Conducting pan-cancer analysis of gene families allows for a holistic understanding of shared genetic alterations or discerning context-specific variations, uncovering divergent roles across cancer types and informing targeted therapeutic strategies tailored to the unique characteristics of individual genes within the family^30,31^. We also conducted a comprehensive pancancer analysis of kindlin genes using data from the TCGA, COSMIC, and ICGC databases across 33 cancer types (Supplementary Note 1)^32–34^. We employed structural and functional genomic tools to investigate the influence of Kindlin family proteins on mechanochemical signaling in various cancers. Our results highlight the role of kindlins in processes related to tumor progression, metastasis, and epithelial–mesenchymal transition, suggesting that they participate in essential mechanosensitive pathways. Furthermore, our study suggested a potential link between kindlin dysfunction and adverse survival outcomes. Utilizing normal mode analysis (NMA), we predicted how cancer-specific mutations in kindlin proteins may impact their stability and flexibility, potentially influencing downstream signaling pathways. This structural genomics approach establishes associations with clinical parameters, providing evidence for the potential mechanochemical importance of kindlins across diverse cancer stages and subtypes.

## Results

### Kindlin alterations are found across multiple cancer types

**Fig. 1:**
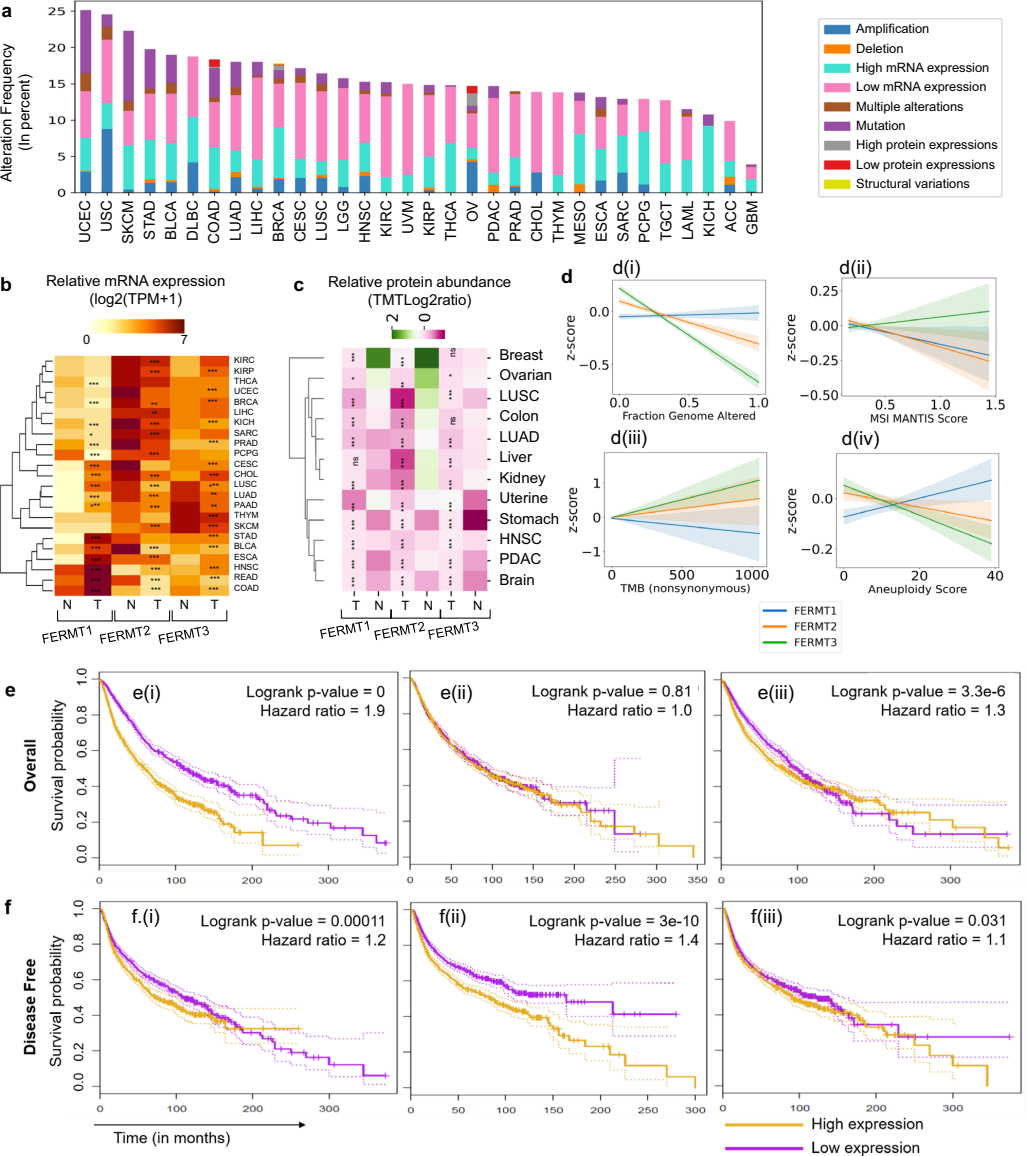
A comprehensive framework of various alterations in the Kindlin family and their impact on prognostic outcomes across different cancer types. **a** Frequency of kindlin alterations in patients with various cancer types. The color codes used in the figure correspond to different types of alterations, as visually depicted. **b** Heatmap representing the comparison of kindlin mRNA expression between normal and tumor samples; the expression scale is log2 (TPM + 1); a darker color represents a more positive mRNA expression level (paired Student’s *t* test *p* < 0.05 indicates significance, *n* = 10953). ***p < 0.0005; **p < 0.005; *p < 0.05; absence of * = no significance; paired pvalues were obtained by Student’s *t* test. **c** Heatmap representing the comparison of kindlin protein expression between normal and tumor samples; purple represents overexpression, and green represents underexpression (paired Student’s *t* test *p* < 0.05 indicates significance; *n* = 1272). ***p < 0.0005; **p < 0.005; *p < 0.05; ns = not significant; paired *p* values were obtained by Student’s *t* test. **d** Correlations of genomic parameters with kindlin mRNA expression z scores. The parameters used were **d**(**i**) the fraction of the genome altered, **d**(**ii**) the MSI MANTIS score, **d**(**iii**) the tumor mutation burden (TMB), and **d**(**iv**) the aneuploidyscores. Corresponding color shadings indicate 95% CI. **e** Kaplan‒Mayer plot of patients in the high and low kindlin mRNA expression sample groups according to comparative quartile (0.75–0.25) for OS. **e**(**i**) FERMT1 (logrank *p* value = 0; hazard ratio = 1.9); **e**(**ii**) FERMT2 (logrank *p* = 0.81; hazard ratio = 1); **e**(**iii**) FERMT3 (logrank *p* value, 3.336 × 10–6; hazard ratio, 1.3). **f** Kaplan–Mayer plot of the comparative quartile (0.75–0.25) disease-free survival in the high and low kindlin mRNA expression sample groups in the case of **f**(**i**) FERMT1 (logrank *p* = 0.00011; hazard ratio = 1.2); **f**(**ii**) FERMT2 (logrank *p* value, 3.01 × 10–10; hazard ratio, 1.4); **f**(**iii**). FERMT3 (logrank *p* = 0.032; hazard ratio = 1.1). For the high quartile, we set the cutoff at 75%, and for the low quartile, we set the cutoff at 25%. The dotted lines on the survival probability curves represent the 95% confidence intervals.

We conducted a pancancer integrative analysis of kindlin alterations using TCGA/ICGA/COSMIC data (Supplementary Fig. 1). Three types of kindlin family genes were significantly altered in 32 different cancer types, with *FERMT1* being the major contributor (29%), followed by *FERMT2* (the kindlin2 gene, 26%) and *FERMT3* (the kindlin3 gene, 20%), for which the z score cutoff was ±1.96 (*p* < 0.05). Kindin alterations can be attributed to either the amplification of *FERMT* genes or changes in their mRNA expression (Fig. 1a). Kindlin expression levels are known to be associated with mechanically regulated cancer invasion and metastasis^35,36^. It is worth considering that kindlins are differentially expressed in normal tissue. In our sample cohort, we found that both FERMT1 were overexpressed in 11 cancer types including CESC, LUAD, STAD, ESCA etc. but underexpressed in 6 cancer types (Fig. 1b). FERMT3 expression is predominantly hematopoietic lineage specific under normal conditions. However, in cancer, it is found to be overexpressed in KIRP, BRCA, CHOL and HNSC (Fig. 1b). Downregulation of FERMT3 is seen lung, pancreatic, and thyroid cancers (Fig. 1b). Interestingly, FERMT2 expression was significantly lower in the tumors than in corresponding normal samples except for SKCM and HNSC (Fig. 1b). *FERMT1* expression increased with increasing stage of cancer progression in BLCA, COAD, LUSC, and STAD (Supplementary Note 2; Supplementary Fig. 2). A stage-specific decrease in *FERMT2* mRNA expression was not pronounced except in renal cancer, LUAD, or BRCA. An increase in *FERMT3* mRNA expression was associated with a significant stage-specific increase only in renal cancer and uveal melanoma (Supplementary Fig. 2). To determine whether the mRNA expression was consistent with the protein abundance, we analyzed the CPTAC pancancer proteome data from TCGA cohort by comparing tumor and tumor-adjacent normal tissue. Kindlin1 protein expression decreases in most cancer samples. However, kindlin3 protein expression exhibited a combination of up-and downregulation in a cancer-specific manner (Fig. 1c). To determine the cause of these differences, we examined the expression levels of the kindlin-associated miRNAs in cancer samples (Supplementary Tables 1, 2, Supplementary Data 1). These results indicate that miRNA-mediated kindlin expression occurs in a cancer-specific manner and suggest feedback-like looping occurs between kindlin expression and miRNA expression. This connection of the kindlin/miRNA axis to cancer progression and chemoresistance is consistent with experimental evidence^37–39^.

These alterations in kindlins are related to the overall genomic alterations in the samples (Fig. 1d(i)–1d(iv)). With increasing mutation frequency in cancer samples, the *FERMT2* and *FERMT3* expression levels increase significantly, while the *FERMT1*expression level shows an inverse trend (Fig. 1d(iii)) similar to that of the MSI MANTIS score (Fig. 1d(ii)). Conversely, the fraction of genome alterations (Fig. 1d(i)) and aneuploidy score (Fig. 1d(iv)) were negatively correlated with *FERMT2* and *FERMT3* but positively correlated with *FERMT1* expression.

We investigated copy number variations (CNVs) in *FERMT* genes across 33 cancer types and observed that, with a few exceptions (LAML, THCA, and PRAD), most cancers exhibitsignificant CNVs, primarily in the form of heterozygous alterations (Supplementary Fig. 3). Specifically, *FERMT1* and *FERMT3* exhibit heterozygous amplification, while *FERMT2* tends toward heterozygous deletion. Additionally, we explored the expression of DNA methylation, a pivotal regulator of Kindlin gene expression, in 14 cancer types (Supplementary Fig. 4). *FERMT2*is hypermethylated, which is particularly notable in KIRP. Conversely, *FERMT1* generally demonstrates hypomethylation across tumor types, except for prominent hypermethylation in BRCA. Interestingly, hypermethylation of *FERMT1* and *FERMT2*is a survival risk marker in various cancers, including LGG, ACC, KIRC, and SARC.

To determine the correlation between kindlin mRNA expression and cancer prognosis, we conducted survival analysis for each kindlin in a pancancer cohort. Elevated *FERMT1* expression was associated with significantly lower overall survival, as indicated by a hazard ratio (HR) of 1.9 (Fig. 1e(i)). Conversely, the expression of *FERMT2* and *FERMT3*did not appear to have a clear connection with overall survival (Fig. 1e(ii), 1e(iii)). Notably, increased *FERMT2* expression may be linked to reduced disease-free survival (Fig. 1f(i)–1f(iii)), suggesting a potential role in chemoresistance or cancer recurrence, as suggested by ref. ^40^ Individual cancer analyses revealed*FERMT1* as a prognostic marker in PAAD (*p* = 0.03, HR = 1.6) and SKCM (*p* < 0.001, HR = 1.7) (Supplementary Fig. 5). A lower*FERMT2* expression may correlate with reduced survival in BLCA (*p* = 0.0036, HR = 1.6) and STAD (*p* = 0.034, HR = 1.4) patients (Supplementary Fig. 6). *FERMT3* overexpression was found to be a prognostic factor for LAML (*p* = 0.001, HR = 2.5), while *FERMT3* underexpression was found to predict the prognosis in SKCM (*p* = 0.0019, HR = 1.52) (Supplementary Fig. 7).

### Kindlin mutations are linked with tumor progression and metastasis

**Fig. 2:**
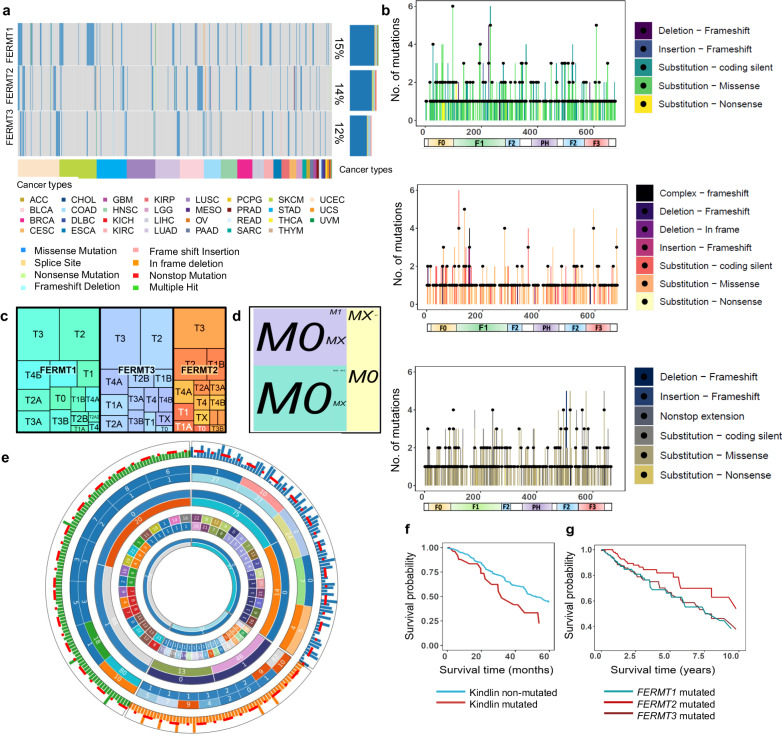
A comprehensive analysis of Kindlin mutations within the TCGA pancancer cohort. **a** The mutation frequency, represented as a percentage of samples, for coding mutations in Kindlin across pancancer samples. Different types of mutations are indicated by distinct colors in the respective patient samples, while the colors at the lower horizontal bar represent the specific types of cancer. **b** The distribution of somatic mutations across all three Kindlins, highlighting the specific types of mutations with corresponding color-coding in patient samples. **c**, **d** Tumor stage-specific and metastatic stage-specific mutations in Kindlins, respectively. The size of each quadrilateral and the corresponding text size for the tumor or metastatic stage indicate the extent of the kindlin alterations involved. Each shade corresponds to different kindlins. **e** Panorama of the effect of noncoding mutations on the kindlin expression profile. Tracks from the inside toward the outside: type of kindlin, cancer type, impact of the mutation, and noncoding mutation type. Comparative plot of *Z* scores for differentially expressed mutated (noncoding) kindlin transcripts (significance cutoff *z* score ± 1.96, *p* < 0.05). Each track consists of two circles, with an inward circle indicating the number of samples per condition and an outward circle indicating the given condition/type. The following conditions are represented by the numbers for each track: track 1 (kindlin name): ‘FERMT1’: 0, ‘FERMT2’: 1; ‘FERMT3’: 2; track 2 (cancer type): Bladder cancer: 0; Blood cancer: 1; Brain cancer: 2; Breast cancer: 3; Breast cancer: 4; Cervical cancer: 5; Colon Cancer: 6; Breast cancer: 7; Endometrial cancer: 8; Gastric cancer: 9; Head and Neck cancer: 10; Liver cancer: 11; Lung cancer: 12; Malignant Lymphoma: 13; Oral cancer: 14; Ovarian cancer: 15; Pancreatic cancer: 16; Pediatric Brain Tumor: 17; Prostate cancer: 18; Rectal cancer: 19; Renal cancer: 20; Renal cancer: 21; Skin cancer: 22; track 3 (mutation type): 3UTR: 0; 5UTR: 1; Downstream: 2; Exon: 3; Intron: 4; Splice Region: 5; Start Gained: 6; Start Lost: 7; Stop Gained: 8; Stop Lost: 9; Upstream: 10; mutation impact: High:For ease of visualization, the expression z scores are rescaled from 0 to 1. The red line passing through the last track indicates a z score = 0. **f** Comparative survival time versus survival probability curve for the kindlin-mutated and nonmutated sample cohorts. (*p* = 0.0003, hazard ratio = 1.932). **g** Comparison of survival versus survival probability curve for patients in the different kindlin-mutated sample cohorts. (Kruskal‒Wallis rank sum *p* value: FERMT1–FERMT2, 0.0058; FERMT2–FERMT3, 0.0002; FERMT1–FERMT3, 0.2945).

As depicted in Fig. 2a, mutation frequencies in Kindlins were identified across 31 cancer classes, with 15%, 14%, and 12% for *FERMT1*, *FERMT2*, and *FERMT3*, respectively. Coding somatic mutations consisted of predominantly missense mutations in various cancers, followed by silent and frameshift mutations (Fig. 2b), distributed throughout their sequences (Fig. 2b). Notably, the FERM domain of *FERMT3*exhibited a notably high mutation frequency. Moreover, *FERMT2*contains a mutational hotspot, particularly within the F1 domain. Analysis of tumor stage-specific mutations revealed a similar trend for all kindlins, with concentrations of mutations occurring at tumor stages T2 and T3 indicating their impact on tumor development rather than onset (Fig. 2c). *FERMT* mutations were also significantly more common in the metastatic M0 stage than in the later stage, suggesting that *FERMT* was involved before metastatic onset (Fig. 2d). We assessed the potential impact of mutations in the regulatory region on mRNA expression by evaluating the extent of loss of function, gain of function, or alteration of function compared to wild-type functionality, inferred from recurrence and multiplicity in tumor samples^41^.

The majority of high-impact noncoding mutations in *FERMT1* and *FERMT2* primarily stemmed from the 5’UTR and upstream sequence (Fig. 2e). These regulatory mutations contribute to a consistent decrease in the expression of *FERMT1* and *FERMT2*. Moreover, *FERMT3*exhibited increased expression (Fig. 2e). Furthermore, the impact of regulatory upstream or downstream mutations across *FERMT3* remains low, in contrast to certain start-lost, stop-lost, stop-gained, splice-region, and intronic mutants that maintain a high impact (Fig. 2e). The Kaplan‒Meier curve demonstrated that *FERMT* mutation was significantly associated with increased survival (*p* = 0.0003, HR = 1.932) (Fig. 2f). Further analysis revealed that the *FERMT1* and *FERMT3* mutations posed almost the same survival risk, which was greater than the *FERMT2* mutation-associated risk (Kruskal‒Wallis rank sum pvalue: *FERMT1-FERMT2*, 0.0058; *FERMT2-FERMT3*, 0.0002; *FERMT1- FERMT3*, 0.2945) (Fig. 2g).

### Mutations affect the structure‒function dynamics of kindlins

**Fig. 3:**
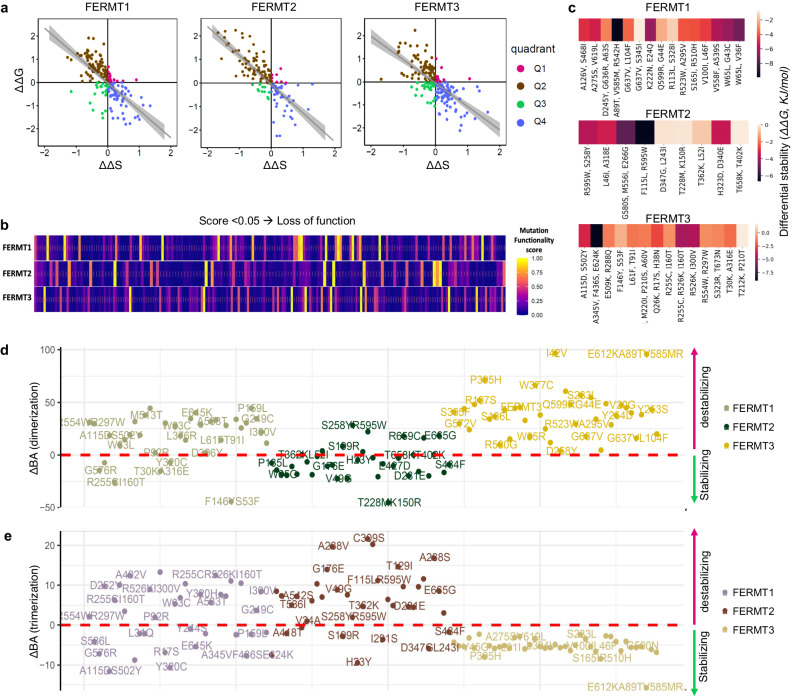
Assessment of the effect of pancancer missense mutations on the physical properties of kindlins, including single or multiple mutation variants. **a** ΔΔG vs. ΔΔS plots to evaluate the stability of the mutants in comparison to their flexibility for Kindlin1 (FERMT1), Kindlin2 (FERMT2), and Kindlin3 (FERMT3). The ΔG and ΔΔS values are provided in units of kJ/mol and unitless, respectively. The plots were divided into four quadrants, namely, Q1, Q2, Q3, and Q4, each corresponding to a different mutant population. Q1, represented in pink, signifies an increase in both stability and flexibility. Q2, shown in brown, indicates an increase in stability but a decrease in flexibility. Q3, marked in green, denotes a decrease in both stability and flexibility. Q4, represented in blue, suggested a decrease in stability but an increase in flexibility. Regression lines with 95% confidence intervals are displayed in gray to further illustrate these trends. **b** Pancancer Kindlin mutants were categorized based on their potential impact on functional activity using the SIFT algorithm. Mutations with values less than 0.05 were classified as loss-of-function mutants, while the remaining mutations were considered neutral mutants.**c**.Assessment of the effect of the pancancer monomeric structural variants of kindlins on stability compared with that of the wild type using ΔΔG values measured in kJ/mol. **d** Effect of pancancer mutations on kindlin dimerization affinity compared to that of wild-type structures; calculated as ΔBA_dimerization_ (KJ/mol) and plotted for highly stabilizing or destabilizing mutants. **e** Effect of pancancer mutations on kindlin trimerization affinity compared to that of wild-type structures; calculated as ΔBA_trimerization_ (KJ/mol) and plotted for highly stabilizing or destabilizing mutants.

Kindlin isoforms exhibit ~54% sequence identity and ~70% sequence similarity, but they are structurally similar (Supplementary Fig. 8). To study the effects of these mutations on the structural stability of kindlins, we calculated the ΔΔG values of all the cancer-specific mutated conformations of all kindlin types. Since the mechanochemical activity of kindlins comes from their domain-specific flexibility, we calculated the change in vibrational entropy (ΔΔS) of the mutants relative to that of the wild-type version. Our analysis revealed four different populations of these mutants: both high flexibility and high stability (Q1), low flexibility and high stability (Q2), both low flexibility and high stability (Q3), and high flexibility and low stability (Q4) (Fig. 3a). We also observed a trend toward decreasing stability with increasing flexibility (*p* < 2.2e-16; σ_FERMT1_ = −0.7470601; σ_FERMT2_ = −0.8190608; σ_FERMT3_ = −0.7077385) (Fig. 3a). Furthermore, we classified the mutants into five categories—very high, high, moderate, low, and slight—for each stabilizing and destabilizing cohort (Supplementary Fig. 9, Supplementary Data 2–4). According to our computational prediction with SIFT^42^, up to 50% cancer-specific mutants of all kindlins might be loss-of-function or pathogenic (Fig. 3b). Most of the loss-of-function mutants were generated from regions with very high stability/low flexibility or very high flexibility/very low stability, i.e., Q2 and Q4, respectively. ΔΔG analysis of multiple mutants also revealed a common destabilizing effect for all the kindlins (Fig. 3c).

All the kindlins showed significant oligomerization ability. However, as the oligomerization of Kindlins has not been fully elucidated^43^, we analyzed both the dimeric (Fig. 3d) and trimeric (Fig. 3e) forms of Kindlins via homology modeling. Changes in the oligomerization properties of the mutants are also evident from the prediction of dimerization and trimerization affinities. Kindlin2 significantly gained dimerization affinity in almost all cancer-specific mutants but lost trimerization affinity in all the other mutants (Fig. 3d, e). In contrast, kindlin3 mutants exhibit more stable trimerization but weaker dimerization than the corresponding mutants (Fig. 3d, e). On the other hand, kindlin1 mutation had a mixed effect on dimerization and trimerization, suggesting altered kindlin-oligomer functionalities (Fig. 3d, e).

Phosphorylation is an important aspect of kindlin functionality and has been validated experimentally at the T8 and T30 positions for kindlin1; at the Y193, S159, S181, and S666 positions for kindlin2; and at the T482 and S484 positions for kindlin3^44^. Computational predictions employing a support vector machine-based machinelearning algorithm on 3D mutated structures^45^ indicated complete loss of the T8 and S484 mutation sites in kindlin1 and kindlin3, respectively. For *FERMT2*, all the frameshift mutants showed complete loss of the Y193 and S666 phosphorylation sites (Supplementary Tables 3–5). These structural effects on phosphorylation correlate with patient-specific phosphorylation levels. According to the phosphor-proteomic tandem mass tag (TMT) data, an overall decrease in phosphorylation was observed for all three kindlins, unlike in *FERMT2*, which suggested that the elevated phosphorylation levels (Supplementary Fig. 10) were plausibly due to altered phosphorylation sites. Overall, this dysregulated phosphorylation plausibly arises due to perturbed kinase activity on these proteins, a signature of tumor cells^46^.

### Kindlins coalter with their interactome to shape the global genomic signature in cancer

**Fig. 4:**
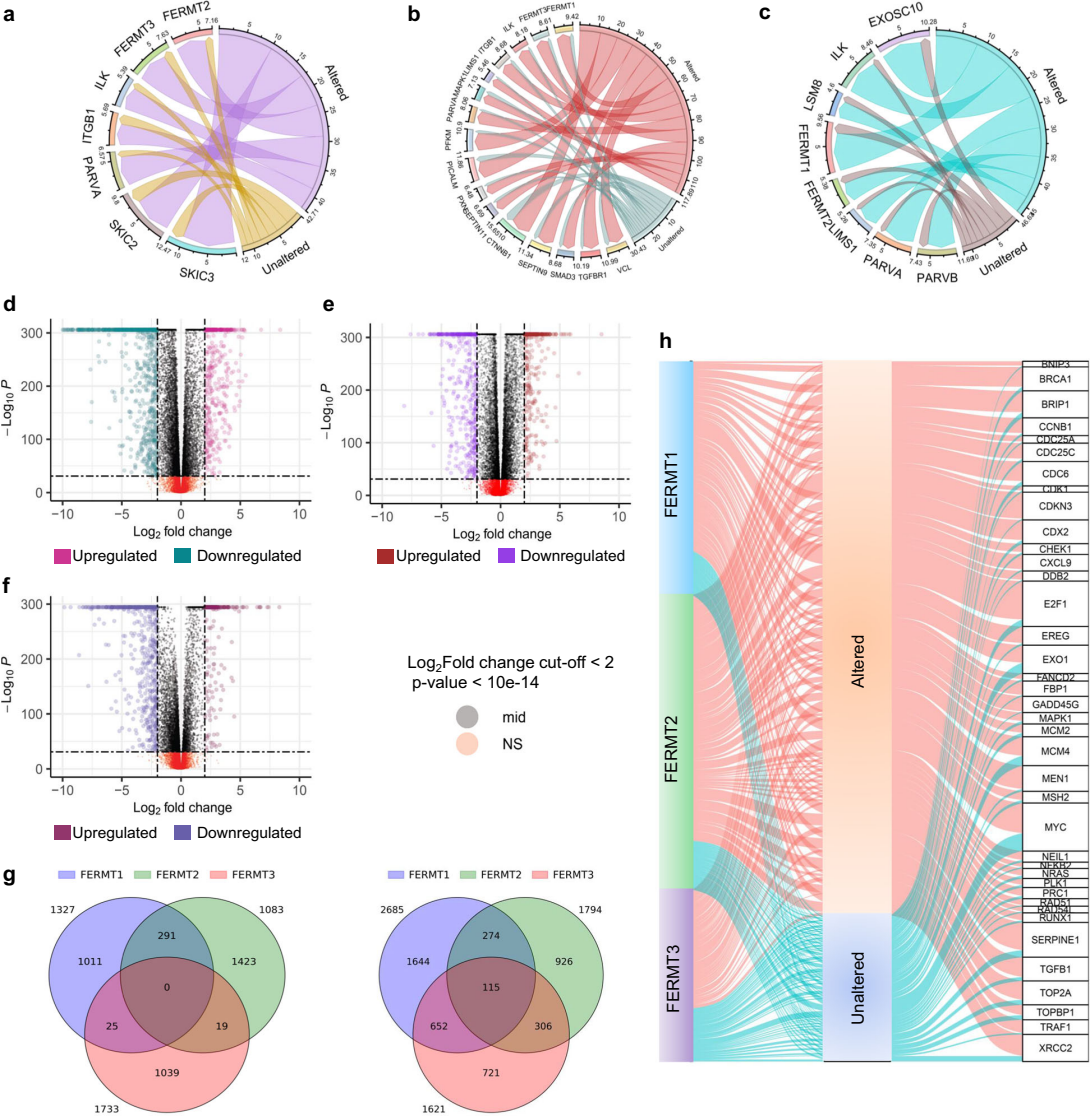
Coalteration analysis revealing global genomic and specific cancerhallmark differences between the kindlin-altered and unaltered cohorts. The alteration frequencies, expressed as percentages, of direct interactors associated with their corresponding Kindlins in both Kindlin-altered and unaltered cancer samples within theTCGA cohort: FERMT1 (**a**), FERMT2 (**b**), and FERMT3 (**c**). The numbers corresponding to each chord represent the percentage of samples in which respective genes are coaltered. Different colored chords are shown for kindlin altered and unaltered samples in each diagram. Differential gene expression analysis of samples with altered expression compared with unaltered expression for Kindlin 1 (**d**), Kindlin 2 (**e**), and Kindlin 3 (**f**). **g** Common and uniquely over-expressed (left) and under-expressed (right) genes shown as Venn diagrams for all three kindlins. **h** Alteration frequencies, represented as percentages, of major cancer hallmark genes from the MsigDB, considering the corresponding kindlin-altered and unaltered cancer samples within the TCGA cohort. The connections between hallmark genes and their status columns are indicated by colored chords, with the width of the chords directly reflecting the number of samples that are either altered or unaltered.

Kindlin subtypes can form massive interaction networks due to their function as adapter proteins to connect many biological processes. Mutations in these proteins can alter their interactions, and their interactors might also be altered in cancer samples, triggering a synergistic effect. Hence, we evaluated the co-alterations of associated interactors in cancer (Fig. 4a–c). The name of the physical interactors corresponding to each kindlin, were identified from STRING and BioGRID database^47,48^. In the *FERMT1*-altered samples, *SKIC3* and *SKIC2*exhibited greater coalterations than did the unaltered samples (Fig. 4a). In the sample cohort with *FERMT2*alterations, we found a similar pattern of coalterations in different partners, including *CTNNB1* and *PFKM* (Fig. 4b). *FERMT3*was mostly coaltered with *EXOSC10* and *ILK* (Fig. 4c). Furthermore, these coalteration behaviors revealed robust mutually exclusive alterations in all the kindlin isoforms (*p* value < 0.001, *q* < 0.001, log2 odds ratio: *FERMT1-FERMT2* > 3, *FERMT2-FERMT3* = 2.77, *FERMT1-FERMT3* = 1.93).

Alterations in all of these kindlins coexist with a global genomic shift. Differential gene expression analysis revealed that many genes were upregulated by kindlin1 and kindlin3 alterations, while fewer genes were overexpressed in the case of kindlin2 (Fig. 4d–f). On the other hand, the number of underexpressed genes was significantly greater in the kindlin2-altered cohort than in the kindlin1 and kindlin3-altered cohort. Interestingly, these kindlin alterations did not result in the expression of any commonly overexpressed genes. In contrast, 115 genes were commonly downregulated in all three kinds of kinase alterations (Fig. 4g). Kindlin2 alterations were associated with the most significant number of uniquely overexpressed genes. In comparison, kindlin1 alterations were associated with the most significant number of uniquely downregulated genes, indicating the differential role of kindlin1 in cancer (Fig. 4g).

Alterations in kindlin levels coincided with significant changes in crucial cancer hallmark genes (Fig. 4h). To measure synergistic effects, we introduced the concept of coalteration dynamics, which represent the average impact of coalterations among the interacting partners in a specific biological process. For instance, we assessed the coalterations of cancer hallmark gene sets related to kindlins, indicating the coalteration dynamics between cancer hallmarks and kindlins. Our analysis, encompassing 39 hallmark genes, revealed the most pronounced coalterations with *FERMT2* (average coalteration dynamics = 14.98), followed by *FERMT1* (average coalteration dynamics = 11.08), with *FERMT3* exhibiting the least coalteration dynamics (average coalteration dynamics = 5.35). This highlights the notable association of kindlin alterations with cancer hallmarks, suggesting their involvement, either directly or indirectly, in promoting cancer.

### Kindlin-related alterations are associated with cancer hallmark pathways

**Fig. 5:**
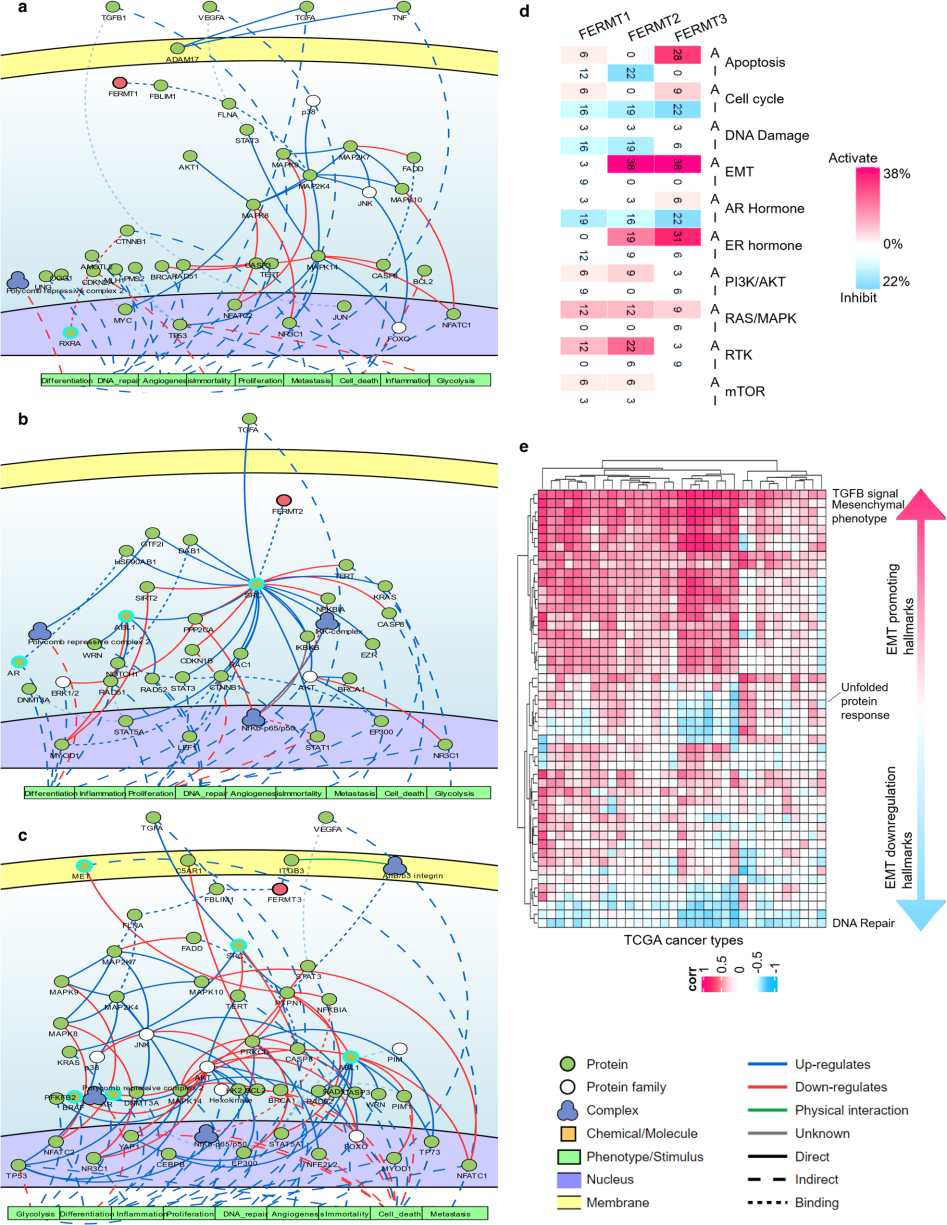
Integrative pancancer modulation of Kindlin-mediated signaling pathways and their role in shaping cancer hallmarks. **a**–**c** Effect of kindlin alterations on pathways contributing to standard cancerhallmarks. The blue arrows denote pathway activation, and the red arrows signify pathway inactivation. **a** Kindlin1; **b** Kindlin2; **c** Kindlin3). **d** The impact of alterations in Kindlins on signature pathways across the TCGA pancancer cohort. These pathways included the cell cycle progression (CCP), DNA damage response (DDR), epithelial–mesenchymal transition (EMT), androgen receptor (AR), estrogen receptor (ER), and receptor tyrosine kinase pathway (RTK). **e** The correlation between Kindlin alterations and EMT hallmarks according to the combination of all three Kindlins. This correlation was measured using the Pearson correlation coefficient.

In our study, we conducted a comprehensive network analysis of Kindlins using CancerGeneNet to explore their impact on nine crucial cancer hallmarks^49^. By analyzing Kindlin1 and Kindlin2 individually, we observed that the alterations in both were associated with essential angiogenesis-activating pathways, such as the TGF, TNF, and VEGF signaling pathways (Fig. 5a, b). Alterations in the expression of these two kindlins cooccur with the inhibition of apoptosis through distinct signaling pathways, including the YAP1, NFkβ, and FOXO pathways. Furthermore, these genes are correlated with the negative regulation of differentiation—Kindlin1 via the polycomb repressor complex/MAP kinase pathway and Kindlin2 through a MYOD1-dependent mechanism (Fig. 5a, b). Kindlin2 alterations are associated with NOTCH1 activation, possibly through enhancing differentiation and cell proliferation via SRC-dependent activation of STATtranscription factors and RAC1 (Fig. 5b). When DNA repair was downregulated, alterations in the expression of both kindlin genes were positively correlated with each other, possibly through the GSK3β pathway or TP53-mediated mechanisms (Fig. 5a, b). Both genes demonstrated associations with promoting immortality through MYC, AKT1, SRC-dependent mechanisms, and telomerase activation (Fig. 5a, b). In the context of metastasis, Kindlin1 was correlated with the activation of the β-catenin and MAPK pathways, while Kindlin2 was correlated with β-catenin and Ezrin activation (Fig. 5a, b). Kindlin1 exhibited a predominant negative correlation with glycolytic pathways in a JNK-dependent manner, whereas Kindlin2 exhibited a positive correlation with glycolysis by influencing hexokinase (HK) and phosphofructokinase (PFKM) (Fig. 5a, b). Although Kindlin3 appears to take a seemingly distinct path in oncogenic signaling, correlations indicated its association with the inhibition of glycolysis by targeting hexokinase, similar to Kindlin1 (Fig. 5a, c). Its alterations correlate with angiogenesis through integrin and TGF signaling, simultaneously cooccurring with cell death in a FOXO- and BCL2-dependent manner. Inhibition of differentiation cooccurs with Kindlin3 alterations, possibly through the DNMT3A, EP300, and CEBP transcription factors or through integrin-PTPN signaling (Fig. 5c). Kindlin3 alterations could also govern DNA repair by deactivating either the ubiquitin ligase complex or DNA polymerase δ through integrin-dependent pathways (Fig. 5c). Furthermore, these alterations might correlate with cancer cell proliferation primarily through integrin-dependent mechanisms involving LATS, PI3K, CREB binding protein, or STAT transcription factors (Fig. 5c).

Apart from the abovementioned genomic level analysis, we examined how Kindlins contribute to ten major cancer-associated pathways at the patient level (Fig. 5d). Similar to *FERMT2* but to a lesser extent, *FERMT1* alterations are associated with the inhibition of apoptosis, cell cycle progression, the DNA damage response, and the androgenreceptor pathway. Moreover, the *FERMT3*-altered patient cohort exhibited major activation of apoptotic pathways. Kindlin-altered patients also exhibited alterations in the PI3K/AKT, mTOR, RTK, MAPK, and hormone signaling pathways. Notably, *FERMT3* and *FERMT2* alterations were significantly associated with epithelial–mesenchymal transition (EMT) (Fig. 5e). Our analysis revealed strong links between EMT-promoting processes, such as UV response downregulation, TGFβ expression, angiogenesis, and hedgehog signaling, and alterations in 33 cancer types. Conversely, EMT-inhibiting pathways, such as DNA repair, oxidative phosphorylation, and P53 tumor suppression pathways, negatively correlate with Kindlin-related alterations. Kindlin alterations also align with EMT-related immune responses, underscoring their role in protecting EMT phenotypes from immunosurveillance (Supplementary Fig. 11).

### Kindlins cooperate with the cancer mechanome and related biological processes

**Fig. 6:**
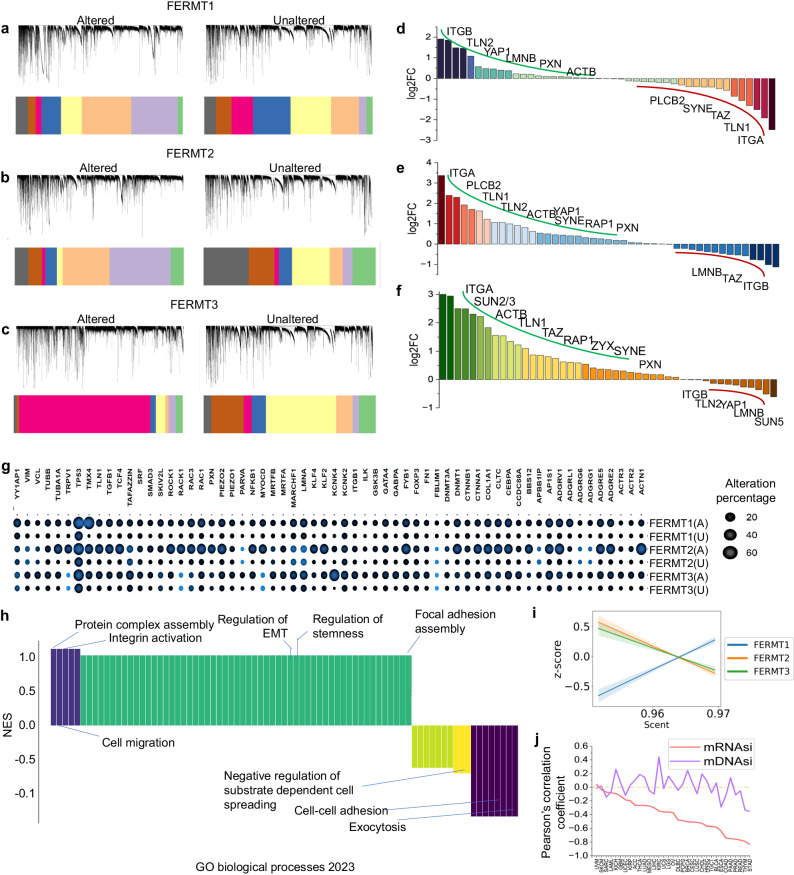
Effect of Kindlin alteration-induced mechanochemical alterations in the pancancer cohort. **a**–**c** Protein‒protein interaction network-based WGCNA of Kindlin altered and unalteredsamples. The clustering dendrogram and expression heatmap of genes identifying the WGCNA modules are shown. Each module is colored separately but modules with similar genes in each plot are depicted using same color. Gene clustering was based on TOM-based dissimilarities. **d**–**f** DEseq analysis of mechanosensitive genes in kindlin-altered samples with respect to unaltered samples for kindlin-1 (**d**), kindlin-2 (**e**), and kindlin-3 (**f**). *p* < 0.005. **g** Comparative analysis of alteration frequencies, represented as percentages, of major proteins associated with the respective kindlins involved in mechanochemical signaling, considering their respective kindlin-altered and unaltered cancer samples from the TCGA cohort. The size of the circles corresponds to the percentage of altered or unaltered samples, while the colors signify the pvalues, which are provided alongside the image. We considered *p* values less than 0.05 to indicate statistical significance. **h** Gene set enrichment analysis of Kindlins in the TCGA pancancer cohort, focusing on GObiological processes. The results are represented by the NES (normalized enrichment score). **i** Correlations between kindlin alterations and cellular potency (scent) in the pancancer cohort. Corresponding color shadings indicate 95% CI. **j** The correlation of the mRNAsi- and mDNAsi-based stemness scores with kindlin alterations in the pancancer cohort.

We used weighted gene coexpression network analysis (WGCNA) to identify modules of genes that exhibited coordinated expression patterns across kindlin-altered samples (Fig. 6a–c)^50^. In cancer, this approach can reveal clusters of genes that work together in specific biological processes or pathways, providing insights into the molecular mechanisms underlying cancer development and progression. We found that kindlinalterations correspond to distinct gene clusters compared to their unaltered counterparts. Although there was no difference in the number of clusters among the kindlins or between the altered and unaltered cohorts (19 for each), the size of the clusters (number of genes in each cluster) varied among the patients. Furthermore, the association among the clustered genes was also altered in kindlin-altered samples compared with control samples, which was also visualized from the 1-TOM-based dendrograms. Interestingly, the clusters of kindlin1 and kindlin2 alterations were somewhat similar, while those of kindlin3 differed in terms of their gene composition (Fig. 6a–c). This helps to identify hub genes within kindlin coexpression modules in cancer, reflecting aimportant role in mechanotransduction and mechanochemical pathways.

In the context of our study, mechanotransduction and mechanochemical pathways were considered two connected but distinct phenomena. Mechanotransduction involves specialized sensors within or on the cell membrane that detect mechanical forces such as tension, compression, shear stress, or stretching. These sensors, exemplified by integrins, then relay signals to trigger various cellular responses. On the other hand, mechanochemical pathways refer to signal modules that can be indirectly influenced by mechanical cues, whether extracellular or intracellular. This category encompasses pathways such as the p53 and mTOR pathways. Kindlins act as interlinks of major cellular pathways, including other mechanosensitive or mechanochemical proteins directly linked to kindlins or through kindlin interactors. Our meta-analysis revealed 53 mechanotransducing proteins of the integrin pathway and 62 mechanochemical proteins, encompassing transcription factors, receptors, ion channels, cytoskeletal proteins, and other types. Differential gene expression analysis revealed that the mechanotransduction protein-encoding genes were most highly expressed when kindlin3 was altered, followed by when kindlin2 was altered (Fig. 6d–f).

On the other hand, in terms of kindlin1 alteration, almost half of these genes were overexpressed, and the other half were downregulated (Fig. 6d). Most of the mechanochemical proteins were coaltered with all the Kindlins (Fig. 6g). Kindlin alterations mostly cooccur with *ACTN1, ADGRs, DNMT1, RAC1, TMX4, and TP53*. Additionally, the mechanochemical protein-forming genes exhibited a very high degree of coalteration with the *FERMT2*gene, followed by that with*FERMT1*, and least common with *FERMT3* (mean coalteration dynamics = 19.3, 12.34, and 9.92, respectively). Most of these coaltered partners were cytoskeletal proteins or transcription factors (Supplementary Data 5).

To determine how kindlin alterations might perturb signaling cascades, we performed pathway enrichment analysis of all the kindlins from the TCGA cohort (Fig. 6h). GO enrichment was found for biological processes and Reactome pathways, as both of these involve the most significant number of updated pathways related to kindlins. We observed that Kindlin alterations in cancer corresponded to highly enriched positive regulation of proteincomplex assembly, cell migration, and integrin activation (Fig. 6h). However, the most negatively enriched pathways were involved in cell aggregation and cell‒cell adhesion, suggesting their role in metastasis (Fig. 6h). Pathway enrichment further revealed greater enrichment of GTPase signaling and reorganization of cellular junctions but a decrease in the cellular response to Ca^2+^ (Fig. 6h).

## Discussion

Precision oncology faces two key challenges: comprehending tumor diversity and predicting changes in intracellular complexity driven by the evolving microenvironment^51,52^. Tumor heterogeneity can lead to chemoresistance and tumor relapse. Recent research has provided a mechanical basis for these events^53,54^. Mechanosensitive adapter proteins, such as kindlins, are vital for connecting external mechanical forces with internal cellular events, functioning like molecular clutches^55^. Any alterations in these proteins can disrupt the cellular balance, potentially fueling malignancy.

This integrative pancancer analysis of kindlin genes was motivated by three major aspects. First, it is imperative to recognize that the Kindlin family comprises multiple closely related proteins (structural similarity ~98%, sequence similarity ~68–73%). However, these genes exhibit a significantly differential expression pattern in different cancers compared to normal tissue. For example, while predominantly known as a hematopoietic lineage-specific protein^56^, Kindlin3 is overexpressed in certain solid cancers, as this finding raises questions about the underlying molecular mechanisms that determine its expression (Fig. 1b). Second, by studying all Kindlin family members collectively, we can gain a comprehensive understanding of their potential complementary and synergistic roles in cancer biology. This includes examining how different Kindlin proteins may interact with each other or with other cellular components to influence cancer cell behavior, tumor progression, and response to therapy. Pathway-specific alteration analysis revealed the combined effect of all the kindlins, especially kindlin2 and kindlin3, on the activation of EMT accompanied by the inhibition of the DNA damage response (Fig. 5d). In addition, kindlins play a role in cellcycle arrest, a common signature of EMT associated with increased ribosome biogenesis^57^. However, it is also interesting how kindlin3 affects cancerhallmark pathways in an integrin-dependent manner, unlike the other two pathways (Fig. 5c). Third, previous studies have proposed a role for kindlins in tumor heterogeneity, chemoresistance, and relapse^40^. These phenotypes are associated with cancer stemness^58^. Our analysis revealed that kindlin expression correlated with decreased potency and hence a plausible role in tumor stem cell differentiation (Fig. 6i). Similarly, cancer stemness was also negatively correlated with kindlin alterations(Fig. 6j). This in turn might cause heterogeneity to drive chemoresistance and relapse^38^, which was also anticipated because high kindlin2 expression causes poor disease-free survival, indicating tumor recurrence (Fig. 1f)^59^. This differentiation is related to EMT promotion and metastasis^60^, both of which are increased in kindlin-altered samples.

The mutational impacts of kindlins are also interesting. Although we found that dimerization mostly stabilizes kindlin2 and destabilizes kindlin3, the opposite trend occurs during trimerization. Kindlin oligomerization is hypothesized to inhibit integrin binding^61^. Considering this, a mixed mutation-specific effect is expected in a patient-specific manner, and to be precise, no trend can be obtained in terms of an oligomerization-dependent effect. However, the overall mutational spectrum indicates altered flexibility and stability and associated mechanochemical alterations. This is further reflected in kindlin-mediated signaling pathways. Our integrative analysis revealed a significant increase in the expression of genes associated with mechanically modulated biological processes such as cell migration, focal adhesion assembly, and cell-matrix interactions (Fig. 6h). The expression of mechanochemical pathway genes was also significantly coaltered with that of all three kindlins, revealing the effect of mechanical perturbations on chemical signals. For example, metabolism in cancer is mechanically regulated^62,63^. We found that, in one instance, kindlin2 activates glycolysis (Fig. 5b); however, it decreases the TCA cycle in an SRC-dependent manner (Supplementary Fig. 12).

The computational data related to kindlin family alterations and mutational and stability analyses presented in our work strongly coincide with those of previous experimental studies. We found that kindlin2 expression is increased in breast cancer and activates epithelial–mesenchymal transition, which was also found by Sossey-Alaoui et al. and Xue et al.^39,64^. It has also been reported that lossoffunction of kindlin2 and kindlin3 leads to cell adhesion deficiency, suggesting the importance of kindlin lossoffunction in their interactions and pathophysiology^65^. Previously, in the case of PAAD, kindlin downregulation was shown to contribute to intratumoral heterogeneity^44^. Based on the nature of the kindlin distribution in normal tissues, we observed genomic alterations in cancers originating from different tissues. These findings led us to propose a plausible role for changes in kindlin family genes in regulating tumor heterogeneity. This heterogeneity corresponds to the activation of different cellular properties within tumor cells. We have shown that kindlin-mediated biochemical alterations arise from combined alterations in kindlins and their networks. Kindlin-mediated cancer-specific upregulation or downregulation of miRNAs can also be important for inducing malignancy and metastasis^37,38^. Our analysis suggested an interesting feedbackloop mechanism involving kindlin and miRNA expression, which has also been shown in breast cancer malignancies^45^. We observed that miRNAs regulated by kindlin2 also target kindlin1 or kindlin3 (Supplementary Table 4). Another interesting observation was the correlation between total mutations and kindlin-1 and kindlin-3 expression levels, as well as the lack of correlation between increases in genomic mutations and kindlin-2 expression. This finding supports the kin-mediated regulation of genomic instability, as was found experimentally by Zhao et al. for breast cancer^46^.

In summary, our analysis unveils the crucial role of mechanosensitive adapter proteins such as kindlins in orchestrating the intricate interplay between external mechanical cues and internal chemical events that drive cancer progression. These alterations likely stemmed from (1) overexpression and heterogeneous amplification; (2) fundamental changes in the structural properties of kindlins, encompassing their stability, flexibility, and force transmission capabilities; and (3) functional characteristics, including phosphorylation events and loss of functional domains. While our findings strongly imply a potential role of kindlins in cancer, it’s vital to stress the need for extensive experimental validation. Most analyses here directly involve patient samples, indicating kindlin involvement in cancer. However, predictive studies, such as mutational effects and identifying loss- or gain-of-function, require validation. Thorough investigations, encompassing structural analyses, biochemical assays, and cellular studies, are essential to validate and contextualize observed associations. Additionally, the novel cancer-specific pathways of kindlins identified in this article warrant further experimental validation. This comprehensive approach will bolster the reliability and depth of our understanding, closing the gap between correlation and causation in the intricate relationship between kindlins and cancer progression. Nonetheless, kindlins are indispensable mechanochemical adaptersthat have substantial influence on a spectrum of cancer signaling pathways. This underscores their potential as promising targets for innovative mechano-modulatory cancer therapeutics, offering context-dependent avenues for intervention and treatment strategies.

## Methods

### Dataset curation and analysis

Preliminary patient RNAseq, CNV, and DNA methylation data were acquired from The Cancer Genome Atlas^32^ (*n* = 10597). Cancer-associated somatic mutations were obtained from the Catalog of Somatic Mutations in Cancer (COSMIC)^34^ (*n* = 24712), and a dataset of multiple mutations per donor was curated from the International Cancer Genome Consortium (ICGC)^33^ database. Along with the kindlin isoform donors (*n* = 1045), mutation-specific expression data (*n* = 268) from 18 cancer cohorts were also found in the ICGC dataset. For the latter, initial data were filtered for donors who possessed high and low functional impact mutations only, leaving out donors with mutations of unknown impact. Comparative Kindlin gene expression analysis of tumor and normal samples was performed using the TCGA cancer cohort and corresponding GTEx normal tissue data, leading to a comparative analysis of 17 cancer types. Finally, kindlin mRNA expression was studied as a function of tumor stage (AJCC) and metastasis stage code (AJCC), which are two clinical attributes for cancer patients. Pancancer protein expression (massspectrometry) data were obtained from the CPTAC dataset for 12 types of cancer. We were able to obtain 1272 and 808 tumor and tumor-adjacent tissue samples, respectively. To analyze the protein expression data, we studied the relative protein abundance as determined by the TMTlog2 ratio. Similarly, for phosphorylation analysis, we obtained tumor and tumor-adjacent data from 1272 and 782 samples, respectively. We estimated the phosphorylation alteration as follows (Eq. 1):

1 $${Log}2{Fold}\,{change}=\frac{{phosphorylation}\,{level}\ ({tumor})}{{phosphorylation}\,{level}\ ({tumor}\,{adjacent}\,{tissue})}$$

Consequently, relevant microRNAs and their expression were analyzed in the context of cancer via systematic analysis of primary literature and textmining of high-throughput experimental data from miRDB (http://www.mirdb.org/)^66^ and miRCancer (http://mircancer.ecu.edu/)^67^ to determine the differences between mRNA expression and protein expression patterns.

### Survival analysis

We used Kaplan‒Meier (KM) plots to analyze overall and disease-free survival based on gene expression. The data were originally sourced from the TCGA/ICGC cohort (*n* = 9498) and utilized in the KM plotter OF GEPIA survival analysis tool^68^. To ensure accuracy, we excluded samples (*n* = 193) that overlapped. For mutation status-specific overall survival plots, we employed data from the TCGA study (*n* = 2583), for which mutation profiles were available.

### Mutated variant analysis

COSMIC data were separately fetched for all the kindlin isoforms, and their pointmutation data were obtained from The Cancer Genome Atlas (TCGA) and International Cancer Genome Consortium (ICGC) databases. Overlapping samples were excluded, for a total of 981 samples were estimated for the analysis.From the consensus transcript sequences of all kindlin isoforms, multiple mutations per transcript of all these isoforms were also documented, with *n* = 38. These multiple-nucleotide variants (MNVs) were further analyzed and filtered manually exhaustively looking for variants that appear- a. in the same individual, b. in the same haplotype, c. in cis position, d. within the window size of 2 basepairs^69^. The functional impact of the single-nucleotide mutations was assessed by the Sorting Intolerant from Tolerant (SIFT) algorithm^42^.

### Cancer-specific mutational stability characterization

The monomeric structures ofkindlin1 (Q9BQL6), kindlin2 (Q96AC1), and kindlin3 (Q86UX7) were derived from AlphaFold^70^. Dimers and trimers were prepared for each of the probes by performing homology modeling on trRosetta^71^. Structural homology was further checked using POSA^72^. Substitution and frameshift mutations were incorporated using v. 2.5.2 PyMOL and the transformed-restrained Rosetta (trRosetta), respectively.

Perturbations in the dynamics and stability of all the kindlin proteins as a function of mutations were categorized by the Elastic Network Contact Model–based NMA (ENCoM–based NMA)^73^ to estimate the change in stability (ΔΔG) and entropy (ΔΔS). The ENCOM-based NMA approach takes into consideration the distinct nature and consequent effects of specific amino acids on the dynamics of the structure^59^. Moreover, the involvement of vibrational normal modes and entropic analysis within the NMA method represents an approach to characterizing protein structure dynamics and the effect of mutations^74^.

### Classification of stabilizing and destabilizing mutants

The mutants’ stability, compared to their respective wild-type structures, was determined by calculating changes in free energy (ΔΔG values). We compiled ΔΔG values for all mutants and repeated the data according to the mutation frequency across all cancer types. We created distinct subsets for mutants that stabilized (ΔΔG (+ve)) and destabilized (ΔΔG (−ve)). The entire dataset was classified by ranking mutations based on their ΔΔG values, arranging them in percentiles (Eq. 2).

2 $$P=\left(n/N\right)* 100$$

*n* = number of samples below a particular value; *N* = total number of samples; *P* = percentile of that particular value.

In the stabilizing dataset, we categorized the 0–25 percentile region (P1) as low, the 25–75 percentile (P2) as moderate, and the 75–100 percentile (P3) as high. Conversely, in the destabilizing dataset, we classified the 0–25 percentile region (P1) as high, the 25–75 percentile (P2) as moderate, and the 75–100 percentile (P3) as low. As statistics commonly use the median as the dividing point between the higher and lower halves of a dataset, we calculated the median for the P1 region in the stabilizing group and the P3 region in the destabilizing group, averaging their magnitudes (Eqs. 3 and 4). Similarly, we computed the median for the P3 region in the stabilizing group and the P1 region in the destabilizing group, averaging their magnitudes as well.

3 $${Average}({low})=\left(\left|{{median}\left(P1\right)}_{{stabilizing}}\right|+\left|{{median}\left(P3\right)}_{{destabilizing}}\right|\right)/2$$

4 $${Average}({high})=\left(\left|{{median}\left(P3\right)}_{{stabilizing}}\right|+\left|{{median}\left(P1\right)}_{{destabilizing}}\right|\right)/2$$

Mutants with ΔΔG values below the average for the low category were labeled as slightly stabilizing, while those with ΔΔG values exceeding the negative of the average for the low category were considered slightly destabilizing, as depicted in Supplementary Fig. 9. Consequently, both single nucleotide mutants that stabilize and destabilize were sorted into five categories: very high, high, moderate, low, and very low. For further downstream analysis, we only considered mutations that were highly stabilizing and highly destabilizing.

### Structural analysis of cancer-specific variants

The characteristic deviation of the stability and flexibility of the mutant (mut) variants against the wild-type (WT) counterparts was determined using the ENCoM-based NMA method^74^. In our analysis, ΔΔG and ΔΔS were calculated as follows (Eq. 5):

5 $$\varDelta \varDelta G={\varDelta G}_{{WT}}{{{-}}}{\varDelta G}_{{mut}}{and}\,\varDelta \varDelta S={\varDelta S}_{{WT}}{{{-}}}{\varDelta S}_{{mut}}$$

where a positive value of ΔΔG indicates stabilization and a negative value indicates destabilization. Similarly, for ΔΔS, a positive value indicates increased flexibility, while a negative value indicates decreased flexibility.

Preliminary data collected from all cancer-associated somatic mutations via this method were subsequently filtered to screen for mutations that corresponded to a ΔΔG_ENCoM_ value of ≥+1.24 and ≤−1.24 for further analysis (as highly stabilizing and destabilizing mutants). We used PhoS3D^45^ to examine the effects of the experimental validated kindlin phosphorylation sites on phosphorylated proteins via 3D pdb coordinates.

The effects of cancer-associated substitution mutations on the ability of monomeric Kindlin to form dimeric and trimeric structures were also predicted via symmetric C2 docking^75^ and C3 docking of the monomer, respectively, employing a hybrid algorithm of template-based and ab initio free modeling and docking^75^.The binding affinity (BA, kcal/mol) of dimerization or trimerization was calculated as follows (Eq. 6):

6 $$\triangle {BA}=\triangle {{BA}}_{{Mut}}-\triangle {{BA}}_{{WT}}$$

(+) vs. ΔBAsuggest destabilization and unfavorable, whereas a (−) vs. value indicates stabilization and is favorable for dimerization and trimerization.

### Copy number variation analysis

We gathered copy number variation (CNV) data for *FERMT1, FERMT2*, and *FERMT3* from both the TCGA and ICGC cohorts. In the CNV analysis module, we computed the percentages of various CNV types and assessed their correlation with mRNA expression for each gene within each cancer type. The raw data were processed with GISTICS2.0^76^ to obtain cancer-specific CNV statistics. However, we determined the correlation between CNV and mRNA expression by analyzing raw CNV data alongside gene-specific mRNA expression from individual samples. We categorized the CNV variations into two subtypes: homozygous, which indicates CNV occurring in both chromosomes, and heterozygous, representing CNV occurring on only one chromosome. We obtained percentage statistics based on these CNV subtypes using GISTIC-processed data. The correlation calculations were performed using the raw CNV and mRNA RSEM data.

### Methylation data analysis

Cancer-specific methylation data were obtained from the NCI Genomic Data Commons for 33 cancer types. Among them, only 14 cancer types contained paired tumor vs. normal data for *FERMT1, FERMT2*, and *FERMT3*. A differential methylation analysis was conducted, considering cancers with at least 10 tumor-normal pairs, using Student’s *t* test. The resulting *p* values were adjusted using FDR, with significance considered at FDR ≤ 0.05.

For methylation-specific survival analysis, patient methylation data were combined with overall survival data. Methylation levels were categorized into high and low groups based on the median methylation. Hazard ratios were estimated through Cox regression analysis. If the Cox coefficient >0, high methylation was indicative of worse survival; conversely, low methylation indicated better survivability. The association between mRNA expression and methylation data was assessed by merging the data using TCGA barcodes for each sample. Pearson’s correlation coefficient was employed to test the relationship between paired mRNA expression and methylation. *P* values were adjusted for FDR, with significance defined as FDR ≤ 0.05.

### DEseq and GSEA

We conducted genomic characterization by performing differential gene expression analysis and gene set enrichment analysis utilizing the Python package pyDESEQ2^77^. Our primary aim was to identify sets of genes that exhibited either high or low expression levels under specific experimental conditions. We used The Cancer Genome Atlas (TCGA) data for 33 cancer types. Our experimental conditions involved comparing altered vs. unaltered states for each kindlin gene. This analysis yielded a list of genes that displayed significant differential expression, accompanied by their log2 (fold change) and p value in the altered cohort compared to the unaltered cohort. In cases where not otherwise specified, the cutoff for log2-fold change was set at 1.5. Subsequently, following the generation of a ranked list of differentially expressed genes for each specific comparison of interest, we conducted gene set enrichment analysis (GSEA).

Additionally, we utilized the same dataset and experimental conditions to perform pathway enrichment analysis, leveraging the Python package GSEApy^78^. We employed the GO Biological Process 2023 pathway set, with the permutation type set to ‘phenotype’ and the method set to’signal_to_noise’. The output of this analysis provided us with a list of biological processes, accompanied by their normalized enrichment scores (NES) and adjusted *p* values. The enriched pathways were ranked based on their NES values, considering the processes with a *p*-value less than 0.05 as statistically significant.

### Coalteration analysis

We conducted a coalteration analysis of TCGA patientsample data for all genes within the direct and indirect physical interactomes of *FERMT1, FERMT2*, and *FERMT3*, sourced from BioGRID4.4 (https://thebiogrid.org/). Additionally, we identified Kindlin-associated mechanochemical proteins through a meta-analysis of text-mined articles (Supplementary Data 5), and hallmark genes were sourced from the MsigDB^79^. The coalteration analysis assessed the extent of the coalterations of these mechanosensitive proteins and hallmark genes in the kindlin-altered and unaltered TCGA cohorts, quantified using the term mean coalteration dynamics defined as follows (Eq. 7):

7 $${{{{{\rm{X}}}}}}=\tfrac{\sum A \% -\varSigma U \% }{N}$$

Here, X = mean coalteration dynamics of a gene set; A% = percentage of samples altered; U% = percentage of samples unaltered; N = number of genes in the set.

Pathway alterations were applied for both functional mutations in kindlin-associated genes. The global percentage of pathway activity for a particular pathway and for a particular kindlin was calculated as follows (Eq. 8):

8 $${Global}\,{percentage}=\left(\frac{{No}. \ {of}\,{cancer}\frac{{activation}}{{inhibition}}}{{No}. \ {of}\,{types}\,{of}\,{cancer}}\right)* 100 \%$$

### Cancer-specific pathway alteration analysis

We used reversed-phase protein array (RPPA) data from the TCPA cohort, which consists of samples included in the TCGA cohort. We utilized these data to calculate scores for cancer samples across 32 different cancer types. Our analysis focused on ten key cancer-related pathways, including apoptosis, cell cycle progression, DNA damage response, EMT, hormone ER, hormone AR, TSC/mTOR, RTK, RAS/MAPK, and PI3K/AKT pathways.

To prepare the data, we employed replicate-based normalized (RBN) RPPA data, which were median-centered and further normalized by standard deviation across all samples for each component. This normalization allowed us to obtain relative protein levels, facilitating pathway alteration comparisons. The pathway score is then the sum of the relative protein level of all positive regulatory components minus that of negative regulatory components in a particular pathway^80^.

We followed the same protocol followed by ref. ^81^. We categorized gene expression into two groups based on median expression levels: high and low. To measure the difference in pathway activity scores (PAS) between these groups, we used Student’s *t* test. The resulting *p* values were adjusted for false discovery rate (FDR) with a cutoff of FDR < = 0.05. For a specific gene, Gene X, if PAS X (high) > PAS X (low), it suggests that Gene X activates the pathway. Conversely, PAS X (high) <= PAS X (low) indicates that Gene X inhibits the pathway.

### Weighted gene coexpression network analysis

We utilized the Python package pyWGCNA to perform a weighted gene correlation network analysis^82^. We began by assessing the degree of gene coexpression similarity between two genes, represented as a and b within a given sample i. This similarity was quantified as T_ab_, which corresponds to the absolute value of their correlation coefficient. To further gauge the strength of the correlation between these genes, we applied a power function, resulting in a correlation measure known as M_ab,_ where *M*_*ab*_ = |*T*_*ab*_|^*β*^.

A gradient approach was used to ensure that our analysis remained scale-independent and to assess the average connectivity. This approach involved systematically adjusting the power value (β) across a range from 1 to 20 to identify the optimal β value that would yield a network displaying a high degree of scale independence, exceeding the threshold of 0.80. The optimal β value was used to construct a scale-free network. Next, we transformed the adjacency matrix into a topological overlap matrix (TOM) and computed the corresponding dissimilarity values (1-TOM). We utilized hierarchical average linkage clustering analysis to identify distinct modules within the network, applying dynamic tree cut to the gene dendrogram with specific criteria, including a cutoff height of 0.975 and a minimum module size of 30 genes.

### Meta-analysis of Kindlin-associated Mechanochemical signaling

We conducted a thorough electronic search of research papers using specific terms such as mechanochemical signaling, mechanosensitive transcription factors, mechanosensitive receptors, and mechanochemical ion channels. In Google Scholar, we found 17,800 articles for mechanochemical signaling, 31,400 for mechanosensitive transcription factors, 60,700 for mechanosensitive receptors, and 49,500 for mechanochemical ion channels. In PubMed, we found 417, 325, 1443, and 2241 articles for these terms, respectively. We eliminated duplicate articles and focused on those specifically related to proteins associated with mechanochemical or mechanosensitive signals (Supplementary Fig. 13).

To further refine our analysis, we cross-referenced the included study references and considered proteins mentioned multiple times only once. We shortlisted relevant abstracts and then investigated the association of these proteins with Kindlins using the extended interactome network of FERMT1, FERMT2, and FERMT3 obtained from BioGRID.

We categorized the mechanochemical proteins into two groups: Level-1, which is directly associated with Kindlins, and Level-2, which is associated with Kindlin interactors. This final list includes 20 mechanochemical transcription factors, 4 mechanochemical ion channels, 6 mechanosensitive receptor proteins, 13 mechanosensitive cytoskeletal proteins, and 14 proteins with other functions.

We obtained information about the major cellular processes involving these genes from GeneCards and individually extracted and recorded these data in a predefined form. These data also specify the type of protein, its primary cellular function, and its association with Kindlin (Supplementary Data 5).

### Cancer stemness and potency measurements

We used the machine learning-based protocol of Malta et al. to quantify the stemness of each cancer patient sample^83^. In brief, the mRNAsi algorithm measures the stemness of samples according to the mRNA expression signature, whereas the mDNAsi algorithm measures stemness from the DNA methylation pattern. Stemness-associated tissue potency was measured using the SCENT algorithm^84^. These values were correlated with kindlin expression (*z* score) using a linear regression method.

### Statistics and reproducibility

All the analyses were performed on TCGA pan-cancer dataset (*n* = 10,953) unless mentioned otherwise. For all differential analyses, altered samples (*n* = 3161 (FERMT1); 2910 (FERMT2); 2168 (FERMT3)) were investigated with respect to corresponding kindlin-unaltered samples (*n* = 7792 (FERMT1); 8043 (FERMT2); 8785 (FERMT3)). Number of overexpressed (*n* = 2405 (FERMT1); 2375 (FERMT2); 2376 (FERMT3)) and underexpressed (*n* = 2380 (FERMT1); 2376 (FERMT2); 2376 (FERMT3)) samples were constant for all expression-based analyses. Mutation analyses were performed on all three kindlin mutated samples (*n* = 627) unless mentioned otherwise. Protein expression and phosphorylation analysis were performed *n* = 1272 CPTAC tumor samples and *n* = 808 tumor adjacent normal samples.Statistical analyses were performed using R version4.2.1, R version4.0.3 (http://cran.r-project.org/) and Origin Pro (https://www.originlab.com/). Routine statistical tests were employed to validate the statistical significance of the differences. These methods included the log-rank *p* test and Cox proportionalhazards model for generating Kaplan‒Meier plots with 95% confidence intervals. The Kruskal‒Wallis test was used to compare survival probabilities based on mutations across all Kindlin isoforms. Unpaired two-tailed *t* tests were used for comparing different population groups, while one-way ANOVA and Bonferroni posthoc correction were applied to evaluate statistical significance among various cancer types at a significance level of *p* ≤ 0.05. Furthermore, *Z* scores were calculated to assess gene expression changes, with a cutoff value of ±1.96. We used -log10 (FDR) fold enrichment with an FDR cutoff of ≤0.05. To verify correlations for nonparametric and parametric variables, we employed Spearman’s and Pearson’s correlation coefficients, where values ranged from +1 (highest correlation) to −1 (lowest correlation), with 0 indicating no correlation. Linear regression was used to analyze the relationship between two variables. In cases involving different populations, we determined significant sample numbers using a power test, considering a power level greater than 0.8 tobe adequate.

### Reporting summary

Further information on research design is available in the Nature Portfolio Reporting Summary linked to this article.

### Supplementary information

Supplementary Information

Description of Additional Supplementary Files

Supplementary Data 1

Supplementary Data 2

Supplementary Data 3

Supplementary Data 4

Supplementary Data 5

Reporting Summary

## Data Availability

Pancancer patient-specific omics data files are available from the respective websites of the International Cancer Genome Consortium (ICGC) (https://dcc.icgc.org/) and The Cancer Genome Atlas (TCGA) (https://portal.gdc.cancer.gov/). Normal tissue gene expression data are available at GTEx (https://gtexportal.org/home/). The corresponding TCGA proteomics data can be accessed from CPTAC (https://proteomics.cancer.gov/data-portal, https://proteomic.datacommons.cancer.gov/pdc/). Pancancer kindlin mutation data can be found in the COSMIC database (https://cancer.sanger.ac.uk/cosmic). The miRNA and TCGA alteration data were obtained from the miRDB (http://www.mirdb.org/) and miRCancer (http://mircancer.ecu.edu/), respectively. Secondary data such as those generated during our analysis are available from https://github.com/SML-CompBio/KINDLIN-PANCAN. The source data are also available with this article as Supplementary Data files and from https://github.com/SML-CompBio/KINDLIN-PANCAN.

## Code availability

The respective software and packages used in this study are described with the corresponding methods and their links. The analysis codes utilized in this research are accessible from 10.5281/zenodo.10775631^85^ and https://github.com/SML-CompBio/KINDLIN-PANCAN/tree/publish.
