# Supplementary material for: Pan-cancer analyses suggest kindlin-associated global mechanochemical alterations
Source: Commun Biol. 2024 Mar 28;7:372. doi: 10.1038/s42003-024-06044-5 (PMC10978987; doi:10.1038/s42003-024-06044-5)
Supplement: Supplementary file 1 — Supplementary Information [file 42003_2024_6044_MOESM1_ESM.pdf]

Supplementary Information

Pan-Cancer analyses suggest kindlin-associated global mechanochemical alterations

Debojyoti Chowdhury†*[1], Ayush Mistry†[2], Debashruti Maity[1], Riti Bhatia[2], Shreyansh Priyadarshi[2], Simran Wadan[2], Soham Chakraborty[2], Shubhasis Haldar*[1,2,3]

[1]Department of Chemical and Biological Sciences, S.N. Bose National Centre for Basic Sciences, Kolkata, West Bengal, India 700106

[2]Department of Biological Sciences, Trivedi School of Biosciences, Ashoka University, Sonepat, Haryana, India 131029

[3]Technical Research Centre, S.N. Bose National Centre for Basic Sciences, Kolkata, West Bengal, India 700106

†Contributed equally to this work

*To whom correspondence should be addressed.

Shubhasis Haldar, Email- shubhasis.haldar@bose.res.in

Debojyoti Chowdhury, Email- debojyoti.chowdhury@bose.res.in

Table of Contents

Supplementary Note 1:Cancer Name Abbreviations

Supplementary Note 2: Gene Descriptions (Direct Interactors of Kindlins)

Supplementary figure 1: Dataset Curation Pipeline Facilitating Downstream Analysis.

Supplementary figure 2: Gene Expression Patterns of All Kindlin Family Proteins Across Multiple Tumor Subtypes.

Supplementary figure 3: Copy Number Variation (CNV) Analysis in 33 Cancer Types

Supplementary figure 4: Gene Methylation Pattern Analysis for All Kindlin Genes across 14 Cancer Types

Supplementary figure 5: FERMT1 Expression-Specific Overall Survival (OS) across 33 Cancer Types.

Supplementary figure 6: FERMT2 Expression-Specific Overall Survival (OS) across 33 Cancer Types.

Supplementary figure 7: FERMT3 Expression-Specific Overall Survival (OS) across 33 Cancer Types.

Supplementary figure 8: Sequence Alignment of All Kindlin Family Proteins Along with Structural Superimposition.

Supplementary figure 9: Classification of Kindlin mutations according to their stabilizing or destabilizing effect.

Supplementary figure 10: Comparative kindlin phosphorylation analysis of tumor and adjacent tissues.

Supplementary figure 11: FERMT2 Gene Expression Association with Immune Cell Profiling Signature.

Supplementary figure 12: Kindlin2 as a connecting link between Integrin Outside-in Signaling & Citric Acid Cycle.

Supplementary figure 13: Methodological pipeline for meta-analysis of kindlin-associated mechanochemical signaling.

Supplementary Table 1: List of microRNAs associated with FERMT1 protein expression regulation in cancers and their expression profile. Up, overexpressed; down, underexpressed.

Supplementary Table 2: List of microRNAs associated with FERMT3 protein expression regulation in cancers and their expression profile. Up, overexpressed; down, underexpressed.

Supplementary Table 3: Phosphorylation Status and Scores for Wild Type and Mutated Kindlin1 for Experimental Phosphorylation Sites. Y, phosphorylated; N, nonphosphorylated.

Supplementary Table 4: Phosphorylation Status and Scores for Wild Type and Mutated Kindlin2 for Experimental Phosphorylation Sites. Y, phosphorylated; N, nonphosphorylated.

Supplementary Table 5: Phosphorylation Status and Scores for Wild Type and Mutated Kindlin3 for Experimental Phosphorylation Sites. Y, phosphorylated; N, nonphosphorylated.

Supplementary Note 1:
Cancer Name Abbreviations:

ACC, adrenocortical cancer

BLCA, Bladder urothelial carcinoma

BRCA, breast invasive carcinoma

CESC, cervical squamous cell carcinoma and endocervical adenocarcinoma

CHOL, Cholangial Carcinoma

COAD, colon adenocarcinoma

DLBC, lymphoid neoplasm diffuse large B-cell lymphoma

ESCA, Esophageal Carcinoma

GBM, Glioblastoma Multiforme

HNSC, head and neck squamous cell carcinoma

KICH, Kidney chromophobe

KIRC, Kidney Renal Clear Cell Carcinoma

KIRP, Kidney Renal Papillary Cell Carcinoma

LAML, acute myeloid leukemia

LGG, Brain Lower Grade Glioma

LIHC, liver hepatocellular carcinoma

LUAD, Lung adenocarcinoma

LUSC, Lung squamous cell carcinoma

MESO, Mesothelioma

MSI, microsatellite instability

MSS, microsatellite stable

OV, ovarian serous cystadenocarcinoma

PAAD, Pancreatic adenocarcinoma

PCPG, Pheochromocytoma and Paraganglioma

PRAD, prostate adenocarcinoma

READ, Rectum adenocarcinoma

SARC, Sarcoma

SKCM, Skin cutaneous melanoma

STAD, Stomach adenocarcinoma

TGCT, Testicular Germ Cell Tumors

THCA, thyroid carcinoma

THYM, Thymoma

UCEC, uterine corpus endometrial carcinoma

UCS, uterine carcinosarcoma

UVM, Uveal melanoma

Supplementary Note *2*:

Gene Descriptions (Direct Interactors of Kindlins)

FERMT1, Fermitin family homolog 1 or Kindlin-1 protein is encoded by this gene, which plays a role in cell adhesion and integrin activation.
FERMT2 encodes a protein called Fermitin family homolog 2 or Kindlin-2 that enhances integrin activation mediated by talins and is needed for the assembly of focal adhesions.
***FERMT3*,**a gene coding for a protein called Fermitin family homolog 3 or Kindlin-3, plays a crucial role in cell adhesion in hematopoietic cells.
SKIC2, Superkiller Viralacidic Activity-2 homolog, is a protein-coding gene that codes for an enzyme in humans called Helicase SKI2 W, which is a helicase that has ATPase activity and is part of the SKI complex.
ITGB1, a gene encoding a protein called Integrin beta-1, is involved in the motility of endothelial cells. It also acts as a receptor for various other proteins, such as fibronectin and collagen.
PARVAis a gene coding for a protein called alpha-parvin, which is important in sarcomere organization and smooth muscle cell contraction and angiogenesis.
***SKIC3*,** agene encoding a protein called tetratricopeptide repeat protein 37, is involved in the SKI complex of proteins to form an exosome that is thought to break down abnormal RNA molecules in the cytosol.
ILK codes for a protein called Integrin-linked Protein Kinase that interacts with integrins and regulates signal transduction.
PARVBis a protein-coding gene for beta-parvin, a protein that binds to ILK and plays a role in integrin signaling and cytoskeletal reorganization.
LIMS1, a gene coding for a protein called LIM and senescent cell antigen-like-containing domain protein 1, is responsible for linking beta-integrins to the actin cytoskeleton.
LSM8 encodes a protein called U6snRNA-associated Sm-like protein LSm8, which is part of a complex involved in spliceosome assembly.
***EXOSC10*, a** protein-coding gene that encodes a protein called exosome component 10, is a part of the RNA exosome complex.
PFKM, the gene encoding a protein called ATP-dependent 6-phosphofructokinase, muscle type, is a protein that plays a catalytic role in a step-in glycolysis.
SEPTIN9 encodes a protein named Septin-9, which is speculated to play a role in cytokinesis.
SEPTIN11codes for a protein called Septin-11, which may play a role in the process of cytokinesis.
PICALMis a gene coding for phosphatidylinositol-binding clathrin assembly protein, which is essential in clathrin-mediated endocytosis.
VCL encodes vinculin, a protein that is an F-actin binding protein and is therefore involved in cell-matrix and cell–cell adhesions. Vinculin is also involved in the mechanosensitive ability of the E-cadherin complex.
PXN, a gene coding for paxillin, is a mechanosensitive cytoskeletal protein that plays a crucial role in focal adhesion sites.
MAPK1 encodes a protein called mitogen-activated protein kinase 1, which is an important component of the MAP kinase signal transduction pathway.
SMAD3 encodes a protein called Mothers against decapentaplegic homolog 3, which is an intercellular signal transducer and is transcriptionally modulated that is regulated by other proteins and kinases.
TGFβR1, a protein-coding gene that forms a heteromeric complex with type II TGFβ receptors when bound to TGFβ, facilitates TGFβ signaling transduction from the cell surface to the cytoplasm.
HIF1A encodes the alpha subunit of the transcription factor hypoxia-inducible factor-1, a heterodimer composed of an alpha and beta subunit that plays an essential regulatory role in multiple cellular processes.
EGFR, a protein-coding gene that is widely recognized for its role in cancer, is a transmembrane glycoprotein in the protein kinase superfamily that serves as a receptor for epidermal growth factor family members.

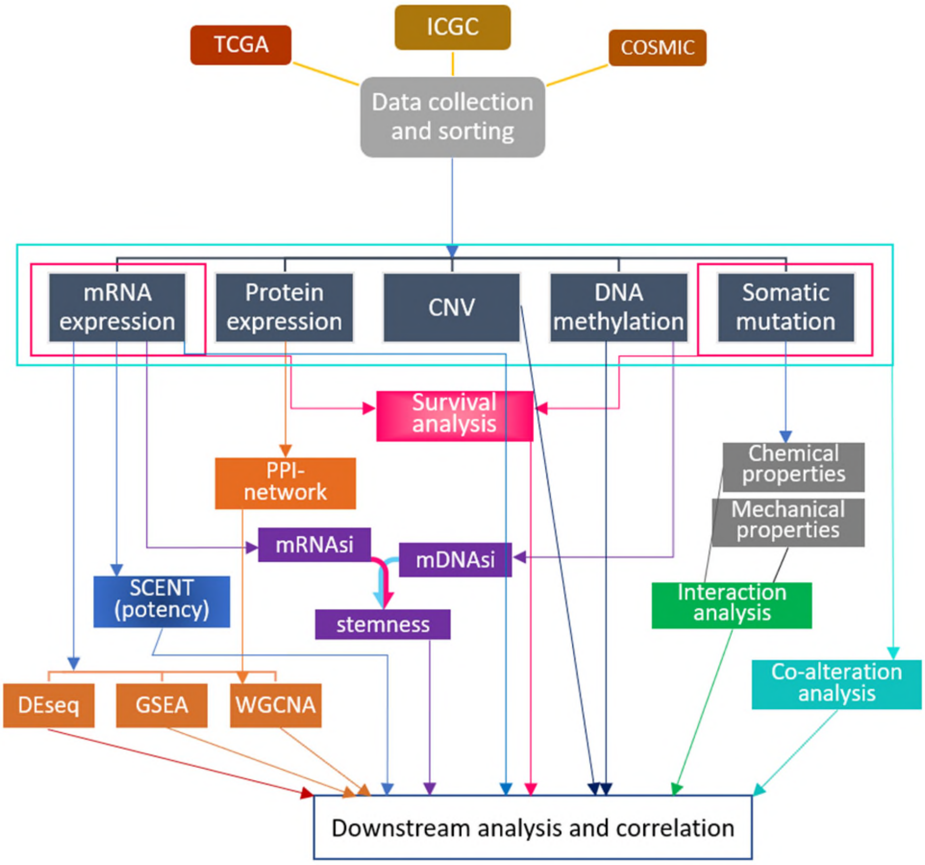

Supplementary figure 1: Dataset Curation Pipeline Facilitating Downstream Analysis. *Data for all Kindlin family proteins, including information related to their expression, somatic mutations, and genomic alterations, were gathered from relevant databases and methodically curated to support specific assays. These assays serve as the fundamental components for subsequent structural and functional analyses.*

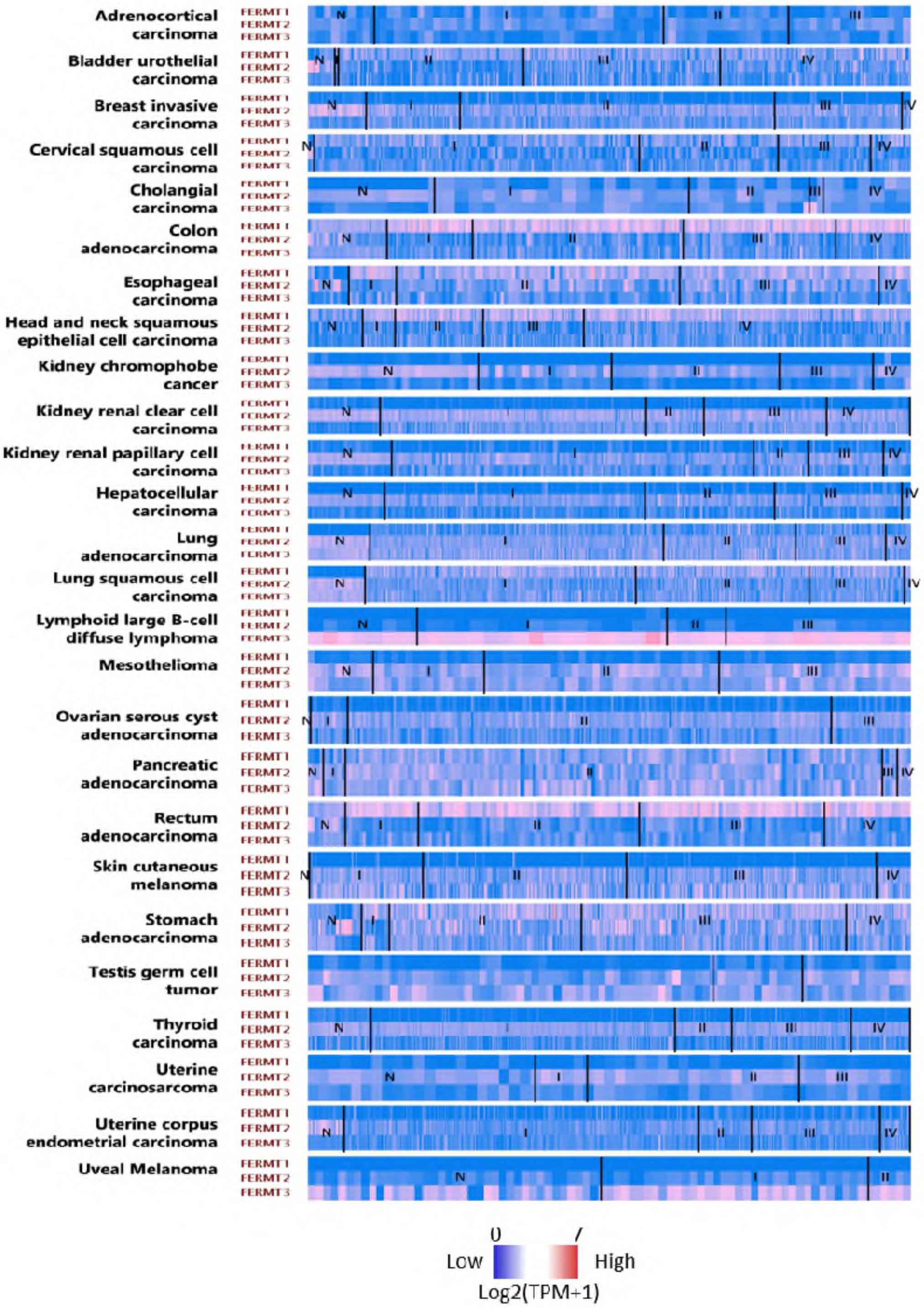

Supplementary figure 2: Gene Expression Patterns of All Kindlin Family Proteins Across Multiple Tumor Subtypes.*The horizontal heatmap comprises individual samples, with the vertical index listing various cancer types and subtypes for the three kindlin proteins. Red indicates elevated expression levels, while blue signifies reduced expression. The heatmap is also subdivided to distinguish between normal tissue (N) and different tumor stages, including stage 1 (I), stage 2 (II), stage 3 (III), and stage 4 (IV). Each of these stages encompasses data for their respective substages and is not further divided to ensure clarity and simplicity of interpretation.*

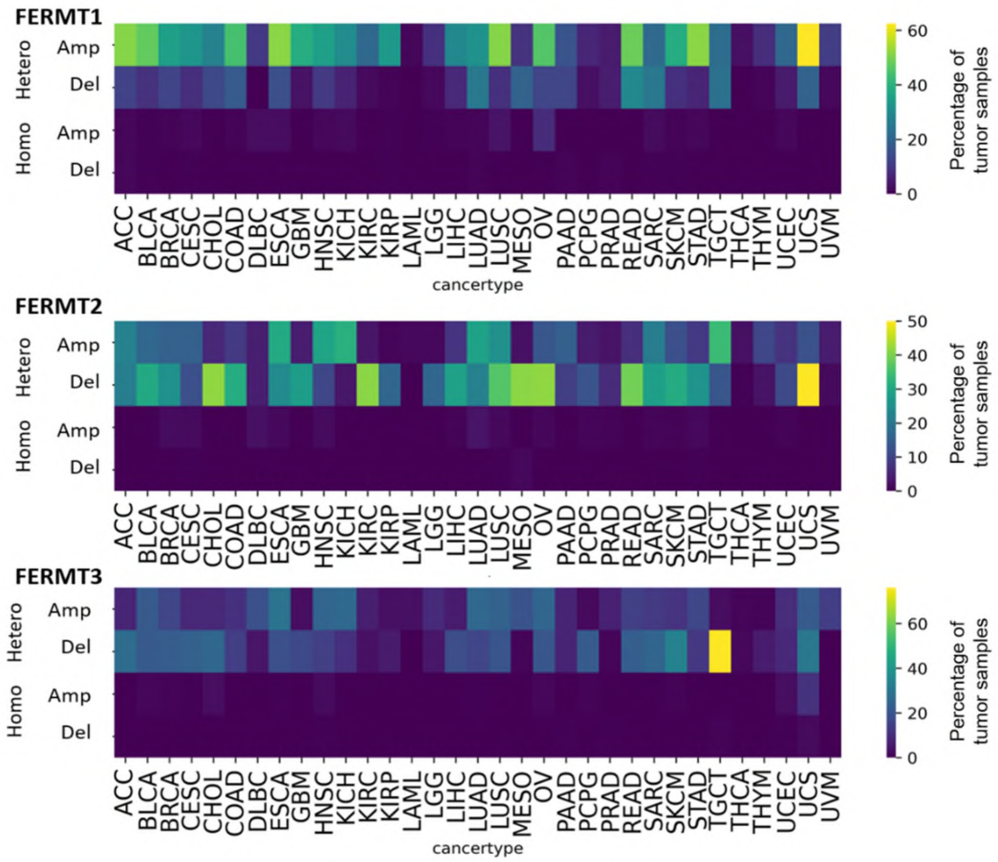

Supplementary figure 3: Copy Number Variation (CNV) Analysis in 33 Cancer Types.*The CNV plot provides a comprehensive view of global copy number variation (CNV) profiles, illustrating the distribution of heterozygous and homozygous CNVs for each kindlin gene in different cancer types. The proportion of each color on the plot reflects the prevalence of that specific CNV type within a given cancer, as indicated by the accompanying scale. Samples with CNV greater than 5% were considered. The colors correspond to different CNV categories: "Amp" for amplification, "Del" for deletion, "Homo" for homozygosity, and "Hetero" for heterozygosity.*

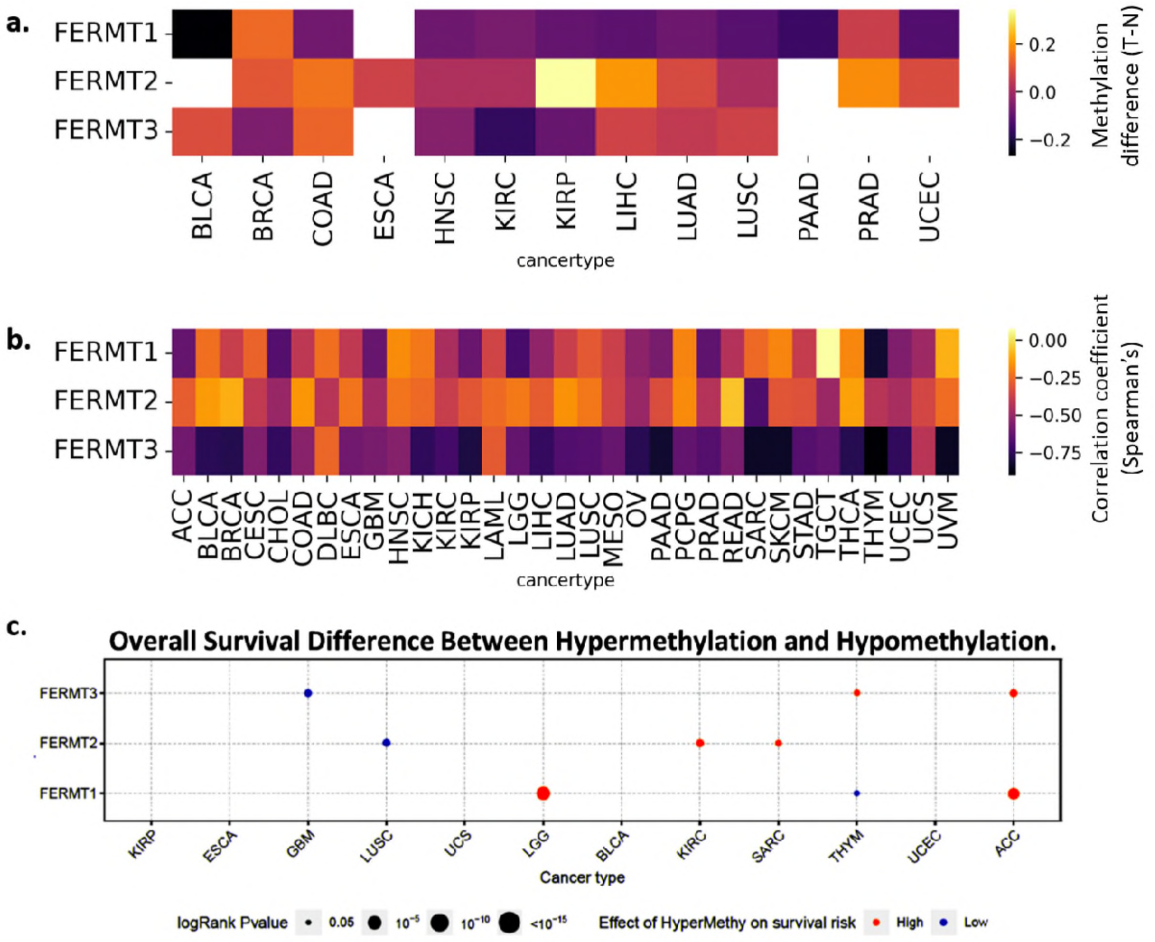

Supplementary figure 4: Gene Methylation Pattern Analysis for All Kindlin Genes across 14 Cancer Types.*a. Differential gene methylation pattern between tumor and normal samples in different cancer types. Purple indicate hypomethylation, whereas orange indicates hypermethylation, both in the case of tumors. b. Genetic correlation between gene methylation and mRNA expression for Kindlin genes derived as Spearman's correlation coefficient. The darkness of colors is representative of a higher magnitude of correlation. c. Survival difference between samples with hyper and hypomethylation of Kindlin genes. The statistical significance of survivability differences was represented using the log-rank p value (≤ 0.05). Red points represent hypermethylation with worse survivability, whereas blue points indicate hypomethylation with better survivability. A larger bubble size is indicative of better significance.*

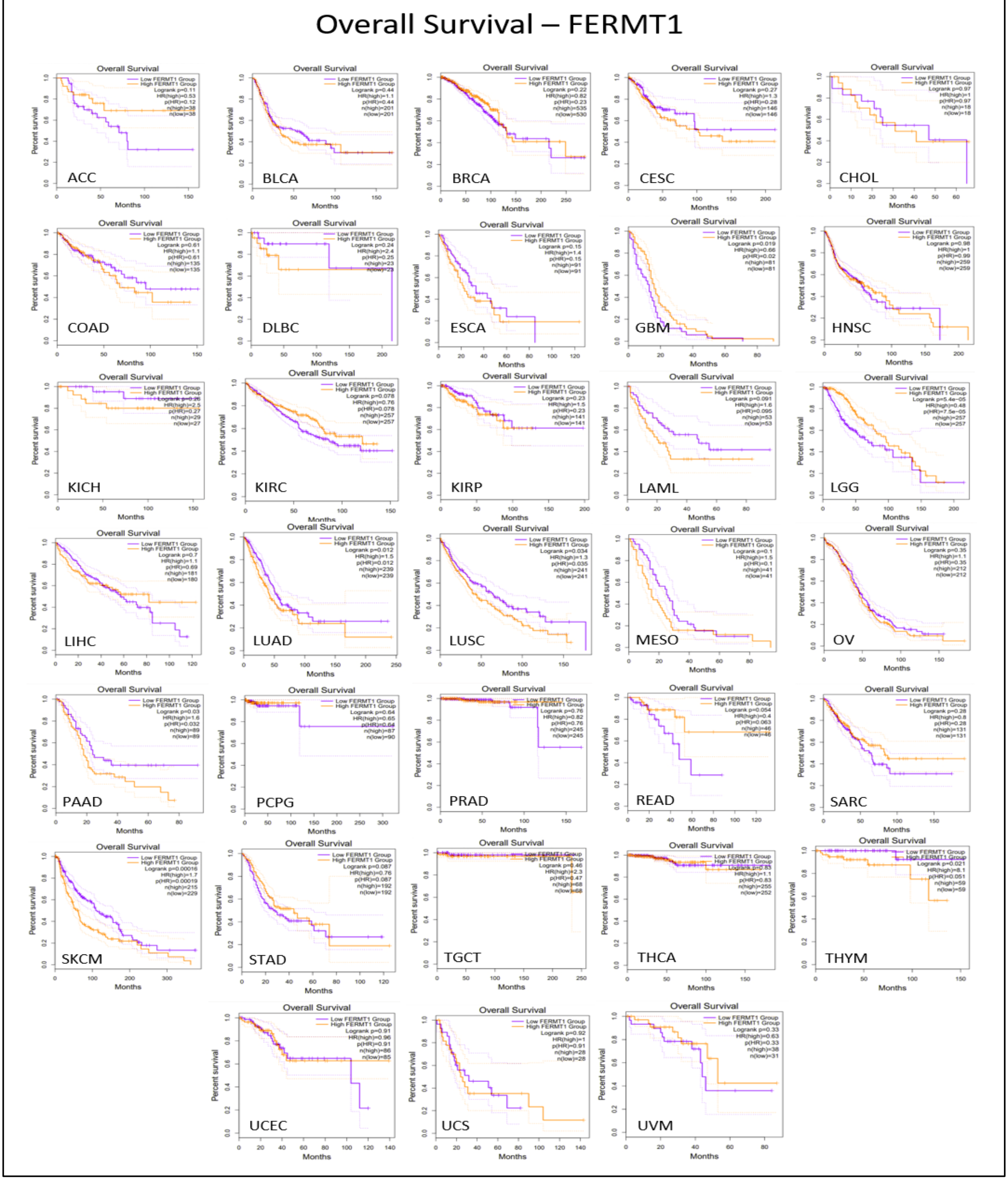

Supplementary figure 5: FERMT1 Expression-Specific Overall Survival (OS) across 33 Cancer Types.*On the x-axis is the time in months since diagnosis, and on the y-axis, the survival probability is shown. Each plot features two lines: a red curve for high FERMT1 transcripts per million and a blue line for low FERMT1 expression levels. A median cutoff was applied in this survival analysis, and 95% confidence intervals are represented by the corresponding dotted lines on the graphs. In the top right corner of each plot, the statistical details, including the log-rank p value, hazard ratio, p value of the hazard ratio obtained through the Cox regression test, and the patient counts in the high and low expressing groups, are mentioned. The respective cancer (sub)-types are labeled in the images.*

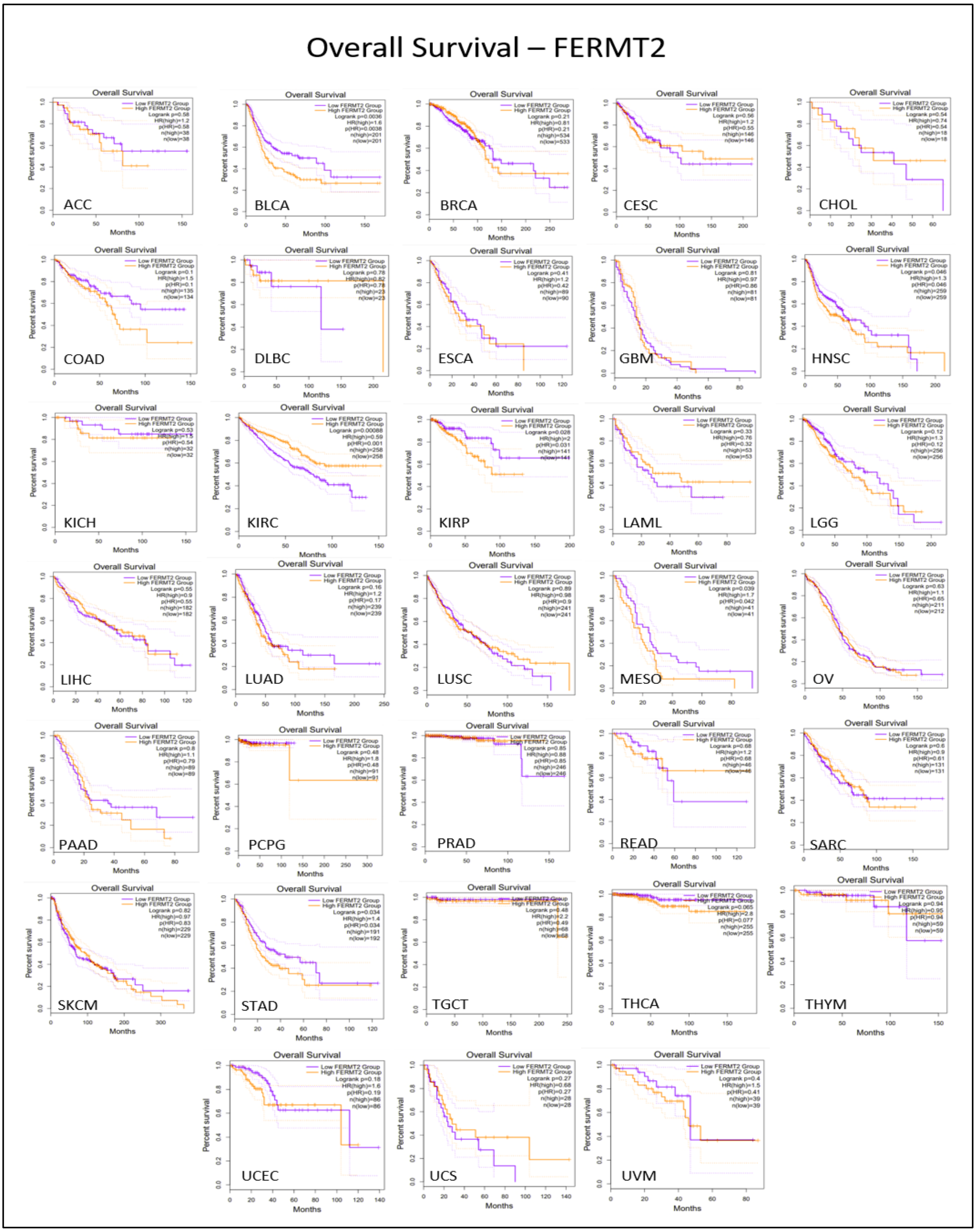

Supplementary figure 6: FERMT2 Expression-Specific Overall Survival (OS) across 33 Cancer Types.*On the x-axis is the time in months since diagnosis, and on the y-axis, the survival probability is shown. Each plot features two lines: a red curve for high FERMT2 transcripts per million and a blue line for low FERMT1 expression levels. A median cutoff was applied in this survival analysis, and 95% confidence intervals are represented by the corresponding dotted lines on the graphs. In the top right corner of each plot, the statistical details, including the log-rank p value, hazard ratio, p value of the hazard ratio obtained through the Cox regression test, and the patient counts in the high and low expressing groups, are mentioned. The respective cancer (sub)-types are labeled in the images.*

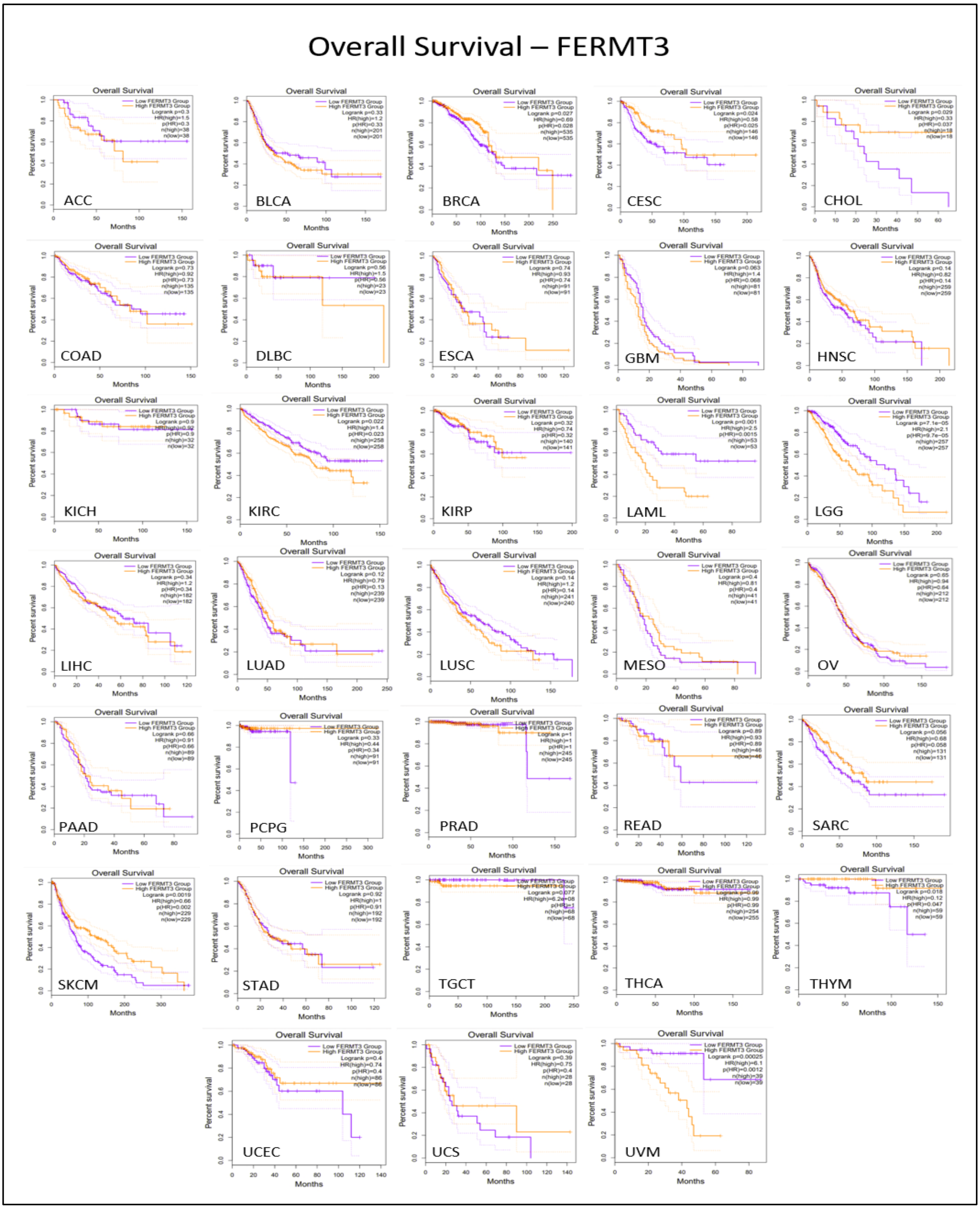

Supplementary figure 7: FERMT3 Expression-Specific Overall Survival (OS) across 33 Cancer Types.*On the x-axis is the time in months since diagnosis, and on the y-axis, the survival probability is shown. Each plot features two lines: a red curve for high FERMT3 transcripts per million and a blue line for low FERMT1 expression levels. A median cutoff was applied in this survival analysis, and 95% confidence intervals are represented by the corresponding dotted lines on the graphs. In the top right corner of each plot, the statistical details, including the log-rank p value, hazard ratio, p value of the hazard ratio obtained through the Cox regression test, and the patient counts in the high and low expressing groups, are mentioned. The respective cancer (sub)-types are labeled in the images.*

a

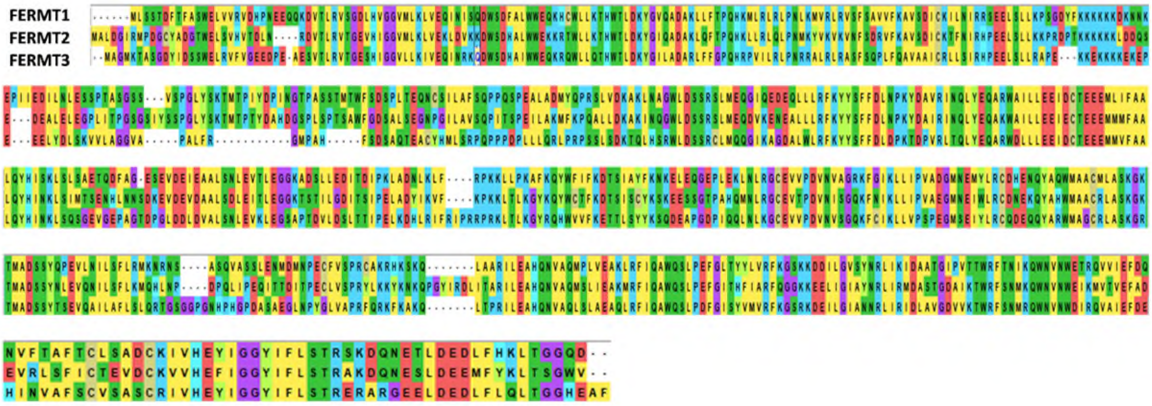

b

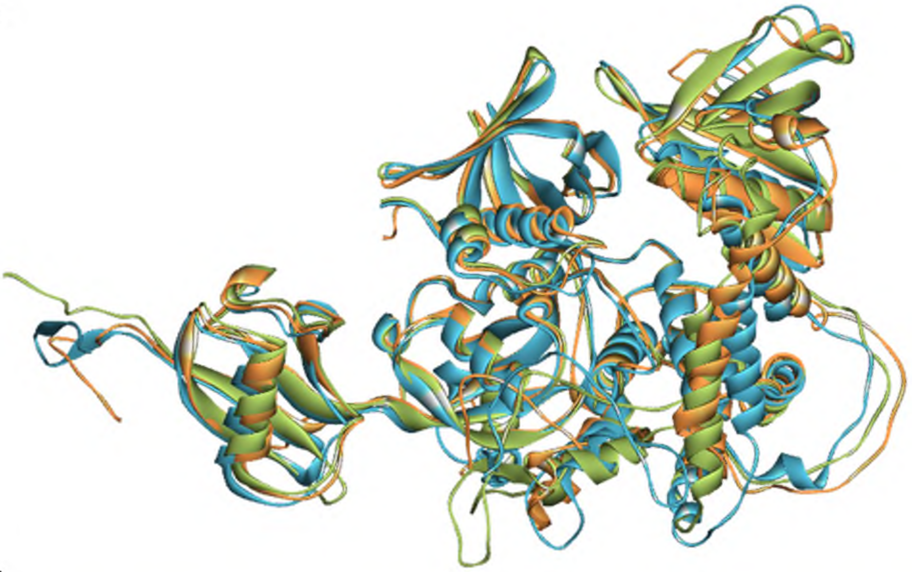

c

#Prot1	Prot2	Superimp-L	RMSD	GL	Iden(%)	Sim(%)
fermt1A-aa.pdb	fermt1A-aa.pdb	677	0.00	0	100.00	100.00
fermt1A-aa.pdb	fermt2A-aa.pdb	656	3.00	33	58.06	73.15
fermt1A-aa.pdb	fermt3A-aa.pdb	644	2.76	40	51.75	68.13
fermt2A-aa.pdb	fermt2A-aa.pdb	680	0.00	0	100.00	100.00
fermt2A-aa.pdb	fermt3A-aa.pdb	643	3.01	50	49.35	67.10
fermt3A-aa.pdb	fermt3A-aa.pdb	667	0.00	0	100.00	100.00

Supplementary figure 8: Sequence Alignment of All Kindlin Family Proteins Along with Structural Superimposition.*a. Multiple sequence alignment of FERMT1, FERMT2, and FERMT3 amino acid sequences. The dots represent gaps with respect to conservativeness. Different colors indicate types of amino acids according to their similarity. Red, acidic; sky blue, basic; yellow, hydrophobic; light green, aromatic; deep green, polar; deep yellow, sulfur-containing; purple, glycine. b. Structural superimposition of Kindlin1, Kindlin2, and Kindlin3 full-length protein monomers. Green, Kindlin1; sky blue, Kindlin2; orange, Kindlin3. c. Protein sequence similarity and identity between different Kindlins. Iden (%), percentage identity; Sim (%), percentage similarity; RMSD, root mean-squared deviation; GL, gap length; Superimp-L, superimposed sequence length.*

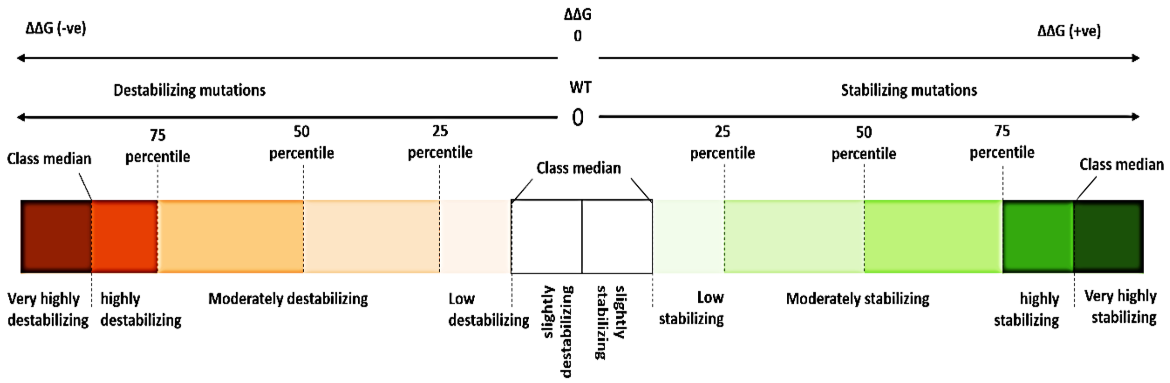

Supplementary figure 9: Classification of Kindlin mutations according to their stabilizing or destabilizing effect. The classifications are based on presently available data. The values (cutoffs) of percentiles and class medians are as follows:

**Status:**	**0th percentile**	**25th percentile**	**50th percentile**	**75th percentile**
**Stabilizing**	0	0.24	0.52	0.89
**Destabilizing**	0	-0.774	-0.416	-0.189

….

Class median	Stabilizing (High)	Stabilizing (Low)	Destabilizing (High)	Destabilizing (Low)
	1.23	0.12	-1.23	-0.12

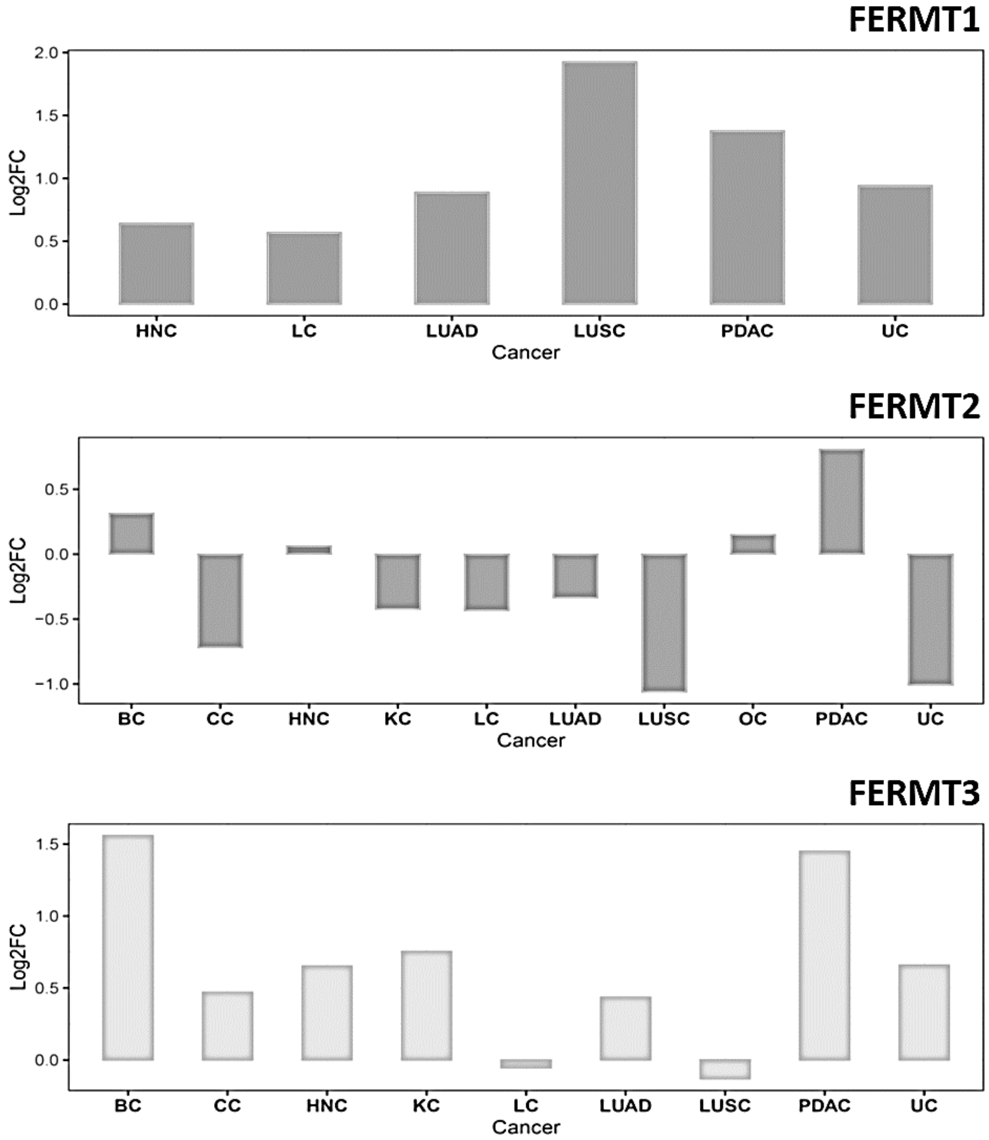

Supplementary figure 10: Comparative kindlin phosphorylation analysis of tumor and adjacent tissues. *Log2-fold change of tumor (n=1272) phosphorylation levels (TMTlog2 ratio) with that of tumor-adjacent tissues (n=782) for all kindlin family proteins from phosphoproteomic data of the CPTAC dataset.*

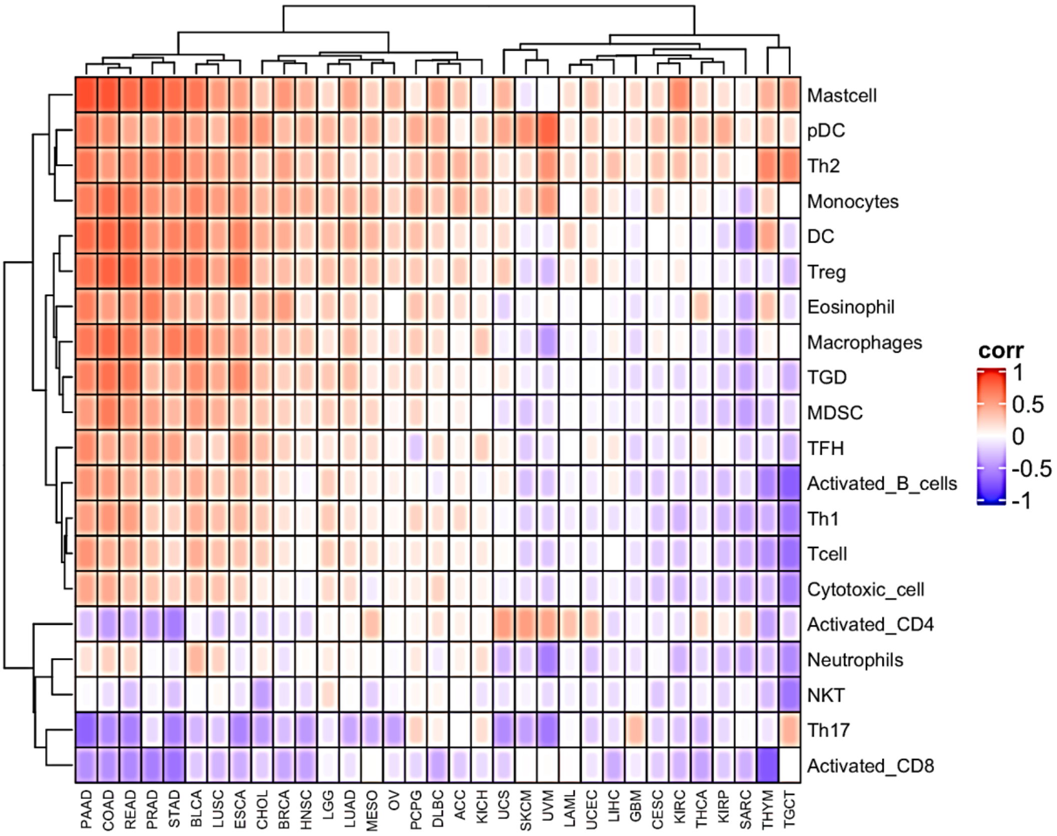

Supplementary figure 11: FERMT2 Gene Expression Association with Immune Cell Profiling Signature.*Colors are indicative of the degree of correlation, where red signifies correlation, and purple signifies anti-correlation. The color intensity is proportional to the extent of correlation or anti-correlation, as represented by the number, where 1 represents the highest correlation and -1 represents the highest anti-correlation.*

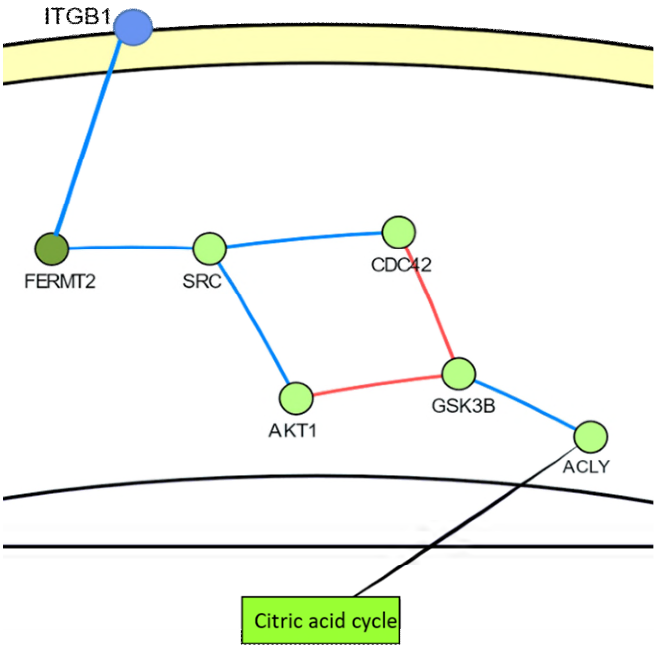

Supplementary figure 12: Kindlin2 as a connecting link between Integrin Outside-in Signaling & Citric Acid Cycle. *The image is taken and modified from CancerGeneNet. Extracellular mechanical signals in cancer cells activate FERMT2 via integrin outside-in signaling. Activated FERMT2 further activates SRC and consequently CDC42 and AKT1 downstream. The activation of CDC42 and AKT1 inhibits GSK3B, which otherwise upregulates the citric acid cycle via ACLY. Blue arrows indicate activation; red lines indicate inhibition; the node distance between FERMT2-SRC = 0.332; SRC-CDC42 = 0.645; SRC-AKT1 = 0.683; CDC42-GSK3B = 0.359; AKT1-GSK3B = 0.834; GSK3B-ACLY = 0.379. The overall Z score through CDC42 = -2.014; through AKT1 = -2.746.*

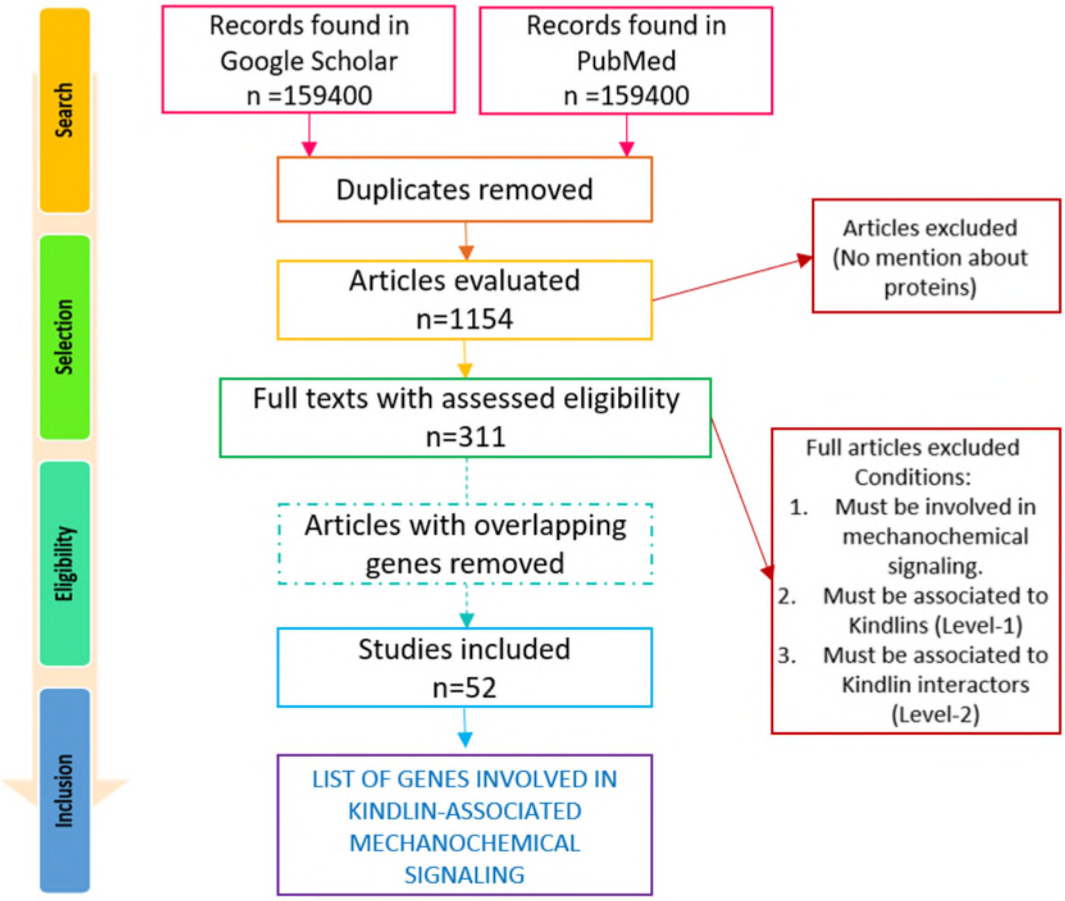

Supplementary figure 13: Methodological pipeline for meta-analysis of kindlin-associated mechanochemical signaling.

Supplementary Table 1: List of microRNAs associated with FERMT1 protein expression regulation in cancers and their expression profile. Up, overexpressed; down, underexpressed.

miRNA	Cancer	Profile
**hsa-miR-186-5p**	Colon	Up
	Osteosarcoma	Down
**hsa-miR-493-3p**	Prostate	Down
**hsa-miR-16-1-3p**	Chronic lymphocytic leukemia	Down
	Gastric	Down
**hsa-miR-5692a**	Hepatocellular	Up
**hsa-miR-9-5p**	Colorectal	Down
	Non-small cell lung	Up
	Pancreatic	Down
	Prostate	Up
**hsa-miR-660-5p**	Breast	Up

	Non-small cell lung	Up
**hsa-miR-567**	Breast	Down
	Osteosarcoma	Down
**hsa-miR-4723-5p**	Prostate	Down
**hsa-miR-577**	Colorectal	Down
	Gastric	Down
	Gastric	Up
	Glioblastoma	Down
	Hepatocellular carcinoma	Down
	Non-small cell lung	Down
	Papillary thyroid carcinoma	Down
**hsa-miR-490-5p**	Bladder	Down
	Hepatocellular carcinoma	Down
	Renal cell carcinoma	Down
**hsa-miR-539-5p**	Glioma	Down

Supplementary Table 2: List of microRNAs associated with FERMT3 protein expression regulation in cancers and their expression profile. Up, overexpressed; down, underexpressed.

miRNA	Cancer	Profile
**hsa-miR-3163**	retinoblastoma	down
**hsa-miR-802**	breast	down
	cervical	down
	gastric	down
	osteosarcoma	up
	prostate	down
**hsa-miR-4775**	colorectal	up
**hsa-miR-542-3p**	astrocytoma	down
	bladder	down
	colorectal	down
	gastric	down
	glioblastoma	down
	hepatocellular carcinoma	down
	malignant melanoma	down
	non-small cell lung	down
	oral squamous cell carcinoma	up
	oral squamous cell carcinoma	down
	osteosarcoma	up
	osteosarcoma	down
**hsa-miR-590-3p**	breast	down
	colon	up
	glioblastoma	down
	glioblastoma	up
	glioma	down
	hepatocellular carcinoma	down
	osteosarcoma	down
	ovarian	up
	prostate	up
**hsa-miR-509-5p**	non-small cell lung	down
	pancreatic	down
	papillary thyroid carcinoma	up
	prostate	down
	renal cell carcinoma	down
**hsa-miR-559**	gastric	down
**hsa-miR-548c-5p**	colorectal	down
**hsa-miR-548a-5p**	hepatocellular carcinoma	up
**hsa-miR-**	gastric	down

**4455**		
**hsa-miR-661**	glioma	down
	non-small cell lung	up
	ovarian	up
	pancreatic ductal adenocarcinoma	up

Supplementary Table 3: Phosphorylation Status and Scores for Wild Type and Mutated Kindlin1 for Experimental Phosphorylation Sites. Y, phosphorylated; N, nonphosphorylated.

Mutation	T8	Score	T30	Score
**FERMT1 WT**	Y	0.641	Y	0.228
**A155D, S502Y**	N	—	Y	0.225
**A244S**	N	—	Y	0.225
**A345V, F436S, E624K**	N	—	Y	0.225
**A402V**	N	—	Y	0.225
**A533T**	N	—	Y	0.225
**D252Y**	N	—	Y	0.225
**D306Y**	N	—	Y	0.225
**E509K, R288Q**	N	—	Y	0.225
**E645K**	N	—	Y	0.225
**F146Y, S53F**	N	—	Y	0.225
**G249C**	N	—	Y	0.225
**G576R**	N	—	Y	0.225
**I124T**	N	—	Y	0.225
**I300V**	N	—	Y	0.225
**L31Q**	N	—	Y	0.303
**L61F, T91I**	N	—	Y	0.223
**L376R**	N	—	Y	0.225
**M477I, P210S, A60V**	N	—	Y	0.225
**M513T**	N	—	Y	0.225
**P92R**	N	—	Y	0.225
**P159L**	N	—	Y	0.225
**Q26K,**	N	—	Y	0.184
**R17S, H38N**				
**R17S**	N	—	Y	0.224
**R255C, I160T**	N	—	Y	0.225
**R255C, R526K, I160T**	N	—	Y	0.225
**R526K, I300V**	N	—	Y	0.225
**R554 W, R297 W**	N	—	Y	0.225
**S133L**	N	—	Y	0.225
**S323R, T673N**	N	—	Y	0.225
**S586L**	N	—	Y	0.225
**T30K, A316E**	N	—	N	—
**T212K, P210T**	N	—	Y	0.225
**W63C**	N	—	Y	0.221
**W63L**	N	—	Y	0.226
**Y235S**	N	—	Y	0.225
**Y244S**	N	—	Y	0.225
**Y320C**	N	—	Y	0.225
**Y320H**	N	—	Y	0.225
**D265EDEL**	N	—	Y	0.227
**F118LDEL**	N	—	Y	0.229
**F206LINS**	N	—	Y	0.227
**N497TDEL**	N	—	Y	0.231
**Q226PINS**	N	—	Y	0.226
**Q226SDEL**	N	—	Y	0.228

**S586HDEL**	N	—	Y	0.23

Supplementary Table 4: Phosphorylation Status and Scores for Wild Type and Mutated Kindlin2 for Experimental Phosphorylation Sites. Y, phosphorylated; N, nonphosphorylated.

**Structure**	Y193	Score	S159	Score	S181	Score	S666	Score
**FERMT2**	Y	0.317	Y	0.079	Y	0.509	Y	0.447
**D57DEL**	N	__	N	__	N	__	N	__
**D459A**	N	__	Y	0.059	Y	0.509	N	__
**E583KDEL**	N	__	Y	0.079	Y	0.407	N	__
**G5DEL**	N	__	N	__	N	__	N	__
**I324DEL**	N	__	Y	0.079	Y	0.44	N	__
**K152DEL**	N	__	N	__	N	__	N	__
**K153INS**	N	__	N	__	N	__	N	__
**K154DEL**	N	__	N	__	N	__	N	__
**K386DEL**	Y	0.317	Y	0.079	Y	0.509	N	__
**L387INS**	N	__	N	__	N	__	N	__
**R290DEL**	Y	0.317	Y	0.079	Y	0.509	N	__
**Y530DEL**	Y	0.317	Y	0.079	Y	0.509	N	__
**A288S**	Y	0.311	Y	0.079	Y	0.509	Y	0.463
**A288V**	Y	0.311	Y	0.079	Y	0.509	Y	0.463
**A418T**	Y	0.311	Y	0.079	Y	0.509	Y	0.463
**A542C**	Y	0.311	Y	0.079	Y	0.509	Y	0.463
**C309S**	Y	0.311	Y	0.079	Y	0.509	Y	0.463
**D281Y**	Y	0.311	Y	0.079	Y	0.509	Y	0.463
**D281E**	Y	0.311	Y	0.079	Y	0.509	Y	0.463
**E427D**	Y	0.311	Y	0.079	Y	0.509	Y	0.463
**E665G**	Y	0.311	Y	0.079	Y	0.509	Y	0.451
**F439C**	Y	0.311	Y	0.079	Y	0.509	Y	0.463
**G176E**	Y	0.311	Y	0.079	Y	0.51	Y	0.463
**H23Y**	Y	0.311	Y	0.079	Y	0.509	Y	0.463
**H323D**	Y	0.311	Y	0.079	Y	0.509	Y	0.463
**I291S**	Y	0.311	Y	0.079	Y	0.509	Y	0.463
**I307T**	Y	0.311	Y	0.079	Y	0.509	Y	0.463
**P135L**	Y	0.311	Y	0.079	Y	0.509	Y	0.463
**R595 W□**	Y	0.311	Y	0.079	Y	0.509	Y	0.463
**R659C**	Y	0.311	Y	0.079	Y	0.509	Y	0.463
**S199R**	Y	0.311	Y	0.079	Y	0.509	Y	0.461
**S484F**	Y	0.311	Y	0.079	Y	0.509	Y	0.463
**T129I**	Y	0.311	Y	0.079	Y	0.509	Y	0.463
**T362K**	Y	0.311	Y	0.079	Y	0.509	Y	0.463
**T536I**	Y	0.311	Y	0.079	Y	0.509	Y	0.463
**V24A**	Y	0.311	Y	0.079	Y	0.509	Y	0.463
**V49G**	Y	0.311	Y	0.079	Y	0.509	Y	0.463
**W65G**	Y	0.311	Y	0.079	Y	0.509	Y	0.463
**Y277D**	Y	0.311	Y	0.079	Y	0.509	Y	0.463

Supplementary Table 5: Phosphorylation Status and Scores for Wild Type and Mutated Kindlin3 for Experimental Phosphorylation Sites. Y, phosphorylated; N, nonphosphorylated.

Mutation	S484	Score	T482	Score
**FERMT3 WT**	N	—	Y	0.454
**F121V**	N	—	Y	0.454
**D258Y**	N	—	Y	0.454
**G500N**	N	—	Y	0.454
**G572V**	N	—	Y	0.454
**G637V**	N	—	Y	0.454
**I42V**	N	—	Y	0.454
**K222N**	N	—	Y	0.454
**L91I**	N	—	Y	0.454
**L304V**	N	—	Y	0.454
**P136L**	N	—	Y	0.454
**P395H**	N	—	Y	0.454
**R107S**	N	—	Y	0.454
**R276Q**	N	—	Y	0.454
**R580G**	N	—	Y	0.454
**S186L**	N	—	Y	0.454
**S232L**	N	—	Y	0.454
**S233L**	N	—	Y	0.454
**S305F**	N	—	Y	0.454
**V20G**	N	—	Y	0.454
**V45G**	N	—	Y	0.454
**W65R**	N	—	Y	0.454
**W377C**	N	—	Y	0.454
**Y253S**	N	—	Y	0.454
**Y254D**	N	—	Y	0.454
**Y557C**	N	—	Y	0.454
**A126V, S468I**	N	—	Y	0.454
**A275S, V619L**	N	—	Y	0.454
**D245Y, G636R, A63S**	N	—	Y	0.454
**E612K, A89T, V585M, R542H**	N	—	Y	0.453
**G637V, S345I**	N	—	Y	0.454
**G637V, L104F**	N	—	Y	0.454
**K222N, E24Q**	N	—	Y	0.454
**Q599R, G44E**	N	—	Y	0.454
**R113L, S328I**	N	—	Y	0.454
**R523 W, A295V**	N	—	Y	0.438
**S165I, R510H**	N	—	Y	0.454
**V100I, L46F**	N	—	Y	0.454
**V558F, A539S**	N	—	Y	0.455
**W65L, G43C**	N	—	Y	0.454
**W65L, V36F**	N	—	Y	0.454
