# Supplementary material for: Pan-cancer analyses suggest kindlin-associated global mechanochemical alterations
Source: Commun Biol. 2024 Mar 28;7:372. doi: 10.1038/s42003-024-06044-5 (PMC10978987; doi:10.1038/s42003-024-06044-5)
Supplement: Supplementary file 2 — Description of Additional Supplementary Files [file 42003_2024_6044_MOESM2_ESM.pdf]

Description of Additional Supplementary Files

File name: Supplementary data File 1

Description: The microRNA data for kndlin2 expression as well as the classification of cancer-associated somatic mutations in all the kindlin isotypes.

File name: Supplementary data File 2

Description: Comparative survival analysis of cancer patients' samples with mutations in three different kindlin isoforms pooled from TCGA.

File name: Supplementary data File 3

Description: The somatic mutations distribution for all three Kindlin and comparative survival time versus survival probability curve for kindlin-mutated and nonmutated sample cohorts.

File name: Supplementary data File 4

Description: Co-alteration analysis revealing global genomic and specific cancer hallmark differences between the kindlin-altered and unaltered cohorts.

File name: Supplementary data File 5

Description: Effect of Kindlin alteration-induced mechanochemical alterations in the pancancer cohort.
